# Structural brain changes in post-COVID condition and its relationship with cognitive impairment

**DOI:** 10.1093/braincomms/fcaf070

**Published:** 2025-02-12

**Authors:** Laura Pacheco-Jaime, Carla Garcia-Vicente, Mar Ariza, Neus Cano, Maite Garolera, Lourdes Carreras-Vidal, Ignacio Roura, Clara Capdevila-Lacasa, Javier Oltra, Jèssica Pardo, Cristina Martín-Barceló, Anna Campabadal, Roser Sala-Llonch, Núria Bargalló, Cristian Barrué, Javier Bejar, Claudio U Cortés, Carme Junqué, Bàrbara Segura, NAUTILUS-Project Collaborative Group, Vanesa Arauzo, Jose A Bernia, Marta Balague-Marmaña, Berta Valles-Pauls, Jesús Caballero, Ester Gonzalez-Aguado, Carme Tayó-Juli, Eva Forcadell-Ferreres, Silvia Reverte-Vilarroya, Susanna Forné, Anna Bartes-Plans, Jordina Muñoz-Padros, Jose A Muñoz-Moreno, Anna Prats-Paris, Inmaculada Rico, Nuria Sabé, Marta Almeria, Laura Casas, Maria José Ciudad, Anna Ferré, Tamar Garzon, Manuela Lozano, Marta Cullell, Sonia Vega, Sílvia Alsina, Maria J Maldonado-Belmonte, Susana Vazquez-Rivera, Eva Baillès, Sandra Navarro, Ayoze González Hernández, Yaiza Molina, Victoria Olive, Silvia Cañizares

**Affiliations:** Medical Psychology Unit, Department of Medicine, Institute of Neurosciences, University of Barcelona, 08036 Barcelona, Catalonia, Spain; Barcelona Clinic Research Fundation - Institute of Biomedical Research August Pi i Sunyer (FRCB-IDIBAPS), 08036 Barcelona, Catalonia, Spain; Medical Psychology Unit, Department of Medicine, Institute of Neurosciences, University of Barcelona, 08036 Barcelona, Catalonia, Spain; Barcelona Clinic Research Fundation - Institute of Biomedical Research August Pi i Sunyer (FRCB-IDIBAPS), 08036 Barcelona, Catalonia, Spain; Medical Psychology Unit, Department of Medicine, Institute of Neurosciences, University of Barcelona, 08036 Barcelona, Catalonia, Spain; Clinical Research Group for Brain, Cognition and Behavior, Consorci Sanitari de Terrassa (CST), 08227 Terrassa, Catalonia, Spain; Clinical Research Group for Brain, Cognition and Behavior, Consorci Sanitari de Terrassa (CST), 08227 Terrassa, Catalonia, Spain; Department of Basic Sciences, International University of Catalonia (UIC), 08017 Sant Cugat del Vallès, Catalonia, Spain; Clinical Research Group for Brain, Cognition and Behavior, Consorci Sanitari de Terrassa (CST), 08227 Terrassa, Catalonia, Spain; Department of Basic Sciences, International University of Catalonia (UIC), 08017 Sant Cugat del Vallès, Catalonia, Spain; Neuropsychology Unit, Consorci Sanitari de Terrassa (CST), 08227 Terrassa, Catalonia, Spain; Medical Psychology Unit, Department of Medicine, Institute of Neurosciences, University of Barcelona, 08036 Barcelona, Catalonia, Spain; Barcelona Clinic Research Fundation - Institute of Biomedical Research August Pi i Sunyer (FRCB-IDIBAPS), 08036 Barcelona, Catalonia, Spain; Medical Psychology Unit, Department of Medicine, Institute of Neurosciences, University of Barcelona, 08036 Barcelona, Catalonia, Spain; Barcelona Clinic Research Fundation - Institute of Biomedical Research August Pi i Sunyer (FRCB-IDIBAPS), 08036 Barcelona, Catalonia, Spain; Medical Psychology Unit, Department of Medicine, Institute of Neurosciences, University of Barcelona, 08036 Barcelona, Catalonia, Spain; Barcelona Clinic Research Fundation - Institute of Biomedical Research August Pi i Sunyer (FRCB-IDIBAPS), 08036 Barcelona, Catalonia, Spain; Medical Psychology Unit, Department of Medicine, Institute of Neurosciences, University of Barcelona, 08036 Barcelona, Catalonia, Spain; Barcelona Clinic Research Fundation - Institute of Biomedical Research August Pi i Sunyer (FRCB-IDIBAPS), 08036 Barcelona, Catalonia, Spain; Aging Research Center, Department of Neurobiology, Care Sciences and Society, Karolinska Institutet and Stockholm University, 171 77 Stockholm, Sweden; Medical Psychology Unit, Department of Medicine, Institute of Neurosciences, University of Barcelona, 08036 Barcelona, Catalonia, Spain; Barcelona Clinic Research Fundation - Institute of Biomedical Research August Pi i Sunyer (FRCB-IDIBAPS), 08036 Barcelona, Catalonia, Spain; Medical Psychology Unit, Department of Medicine, Institute of Neurosciences, University of Barcelona, 08036 Barcelona, Catalonia, Spain; Barcelona Clinic Research Fundation - Institute of Biomedical Research August Pi i Sunyer (FRCB-IDIBAPS), 08036 Barcelona, Catalonia, Spain; Medical Psychology Unit, Department of Medicine, Institute of Neurosciences, University of Barcelona, 08036 Barcelona, Catalonia, Spain; Barcelona Clinic Research Fundation - Institute of Biomedical Research August Pi i Sunyer (FRCB-IDIBAPS), 08036 Barcelona, Catalonia, Spain; Neurology Service, Consorci Corporació Sanitària Parc Taulí, 08028 Sabadell, Catalonia, Spain; Barcelona Clinic Research Fundation - Institute of Biomedical Research August Pi i Sunyer (FRCB-IDIBAPS), 08036 Barcelona, Catalonia, Spain; Department of Biomedicine, Institute of Neuroscience, University of Barcelona, 08036 Barcelona, Catalonia, Spain; Centro de Investigación Biomédica en Red en Bioingeniería, Biomateriales y Nanomedicina (CIBER-BBN), 08036 Barcelona, Catalonia, Spain; Barcelona Clinic Research Fundation - Institute of Biomedical Research August Pi i Sunyer (FRCB-IDIBAPS), 08036 Barcelona, Catalonia, Spain; Centre de Diagnòstic per la Imatge, Hospital Clínic, 08036 Barcelona, Catalonia, Spain; Department of Computer Science, Universitat Politècnica de Catalunya—BarcelonaTech, 08034 Barcelona, Catalonia, Spain; Department of Computer Science, Universitat Politècnica de Catalunya—BarcelonaTech, 08034 Barcelona, Catalonia, Spain; Department of Computer Science, Universitat Politècnica de Catalunya—BarcelonaTech, 08034 Barcelona, Catalonia, Spain; Medical Psychology Unit, Department of Medicine, Institute of Neurosciences, University of Barcelona, 08036 Barcelona, Catalonia, Spain; Barcelona Clinic Research Fundation - Institute of Biomedical Research August Pi i Sunyer (FRCB-IDIBAPS), 08036 Barcelona, Catalonia, Spain; Biomedical Research Networking Center on Neurodegenerative Diseases (CIBERNED), 08036 Barcelona, Catalonia, Spain; Medical Psychology Unit, Department of Medicine, Institute of Neurosciences, University of Barcelona, 08036 Barcelona, Catalonia, Spain; Barcelona Clinic Research Fundation - Institute of Biomedical Research August Pi i Sunyer (FRCB-IDIBAPS), 08036 Barcelona, Catalonia, Spain; Biomedical Research Networking Center on Neurodegenerative Diseases (CIBERNED), 08036 Barcelona, Catalonia, Spain

**Keywords:** post-COVID condition, SARS-CoV-2, cognitive performance, neuroimaging, magnetic resonance imaging

## Abstract

It has been estimated that ∼4% of individuals infected with SARS-CoV-2 will be diagnosed with post-COVID condition. Previous studies have evidenced the presence of cognitive dysfunction and structural brain changes in infected individuals; however, the relationship between structural changes and cognitive alterations in post-COVID condition is still not clear. Consequently, the aim of this work is to study structural brain alterations in post-COVID condition patients after almost 2 years of infection and their likely relationship with patients’ cognitive impairment. Additionally, the association with blood biomarkers and clinical variables was also explored. One hundred and twenty-eight individuals with post-COVID condition and 37 non-infected healthy controls from the Nautilus Project (ClinicalTrials.gov IDs: NCT05307549 and NCT05307575) underwent structural brain magnetic resonance imaging and a comprehensive neuropsychological assessment. A subsample of 66 post-COVID participants also underwent blood extraction to obtain levels of blood biomarkers. Cortical thickness and subcortical volumes were obtained and analysed using FreeSurfer (v7.1). FMRIB Software Library software (v6.0.4) was used to perform grey matter voxel-based analysis and to study microstructural white matter integrity. Patients with post-COVID performed significantly worse in working and verbal memory, processing speed, verbal fluency and executive functions, compared to healthy controls. Moreover, patients with post-COVID showed increased cortical thickness in the right superior frontal and the right rostral middle frontal gyri that negatively correlated with working memory performance. Diffusion tensor imaging data showed lower fractional anisotropy in patients in the right superior longitudinal fasciculus, the splenium and genu of the corpus callosum, the right uncinate fasciculus and the forceps major, that negatively correlated with subjective memory failures. No differences in blood biomarkers were found. Once patients were classified according to their cognitive status, post-COVID clinically cognitively altered presented increased cortical thickness compared to those classified as non-cognitively altered. In conclusion, our study showed that grey and white matter brain changes are relevant in this condition after almost 2 years of infection and partly explain long-term cognitive sequelae. These findings underscore the critical importance of monitoring this at-risk population over time.

## Teaser

This work explores brain and cognitive abnormalities in post-COVID individuals. Pacheco-Jaime *et al*. reported structural brain changes in this condition almost 2 years after infection and partly explain long-term cognitive sequelae. These findings underscore the importance of monitoring at-risk population over time.

## Graphical Abstract

**Graphical Abstract:**
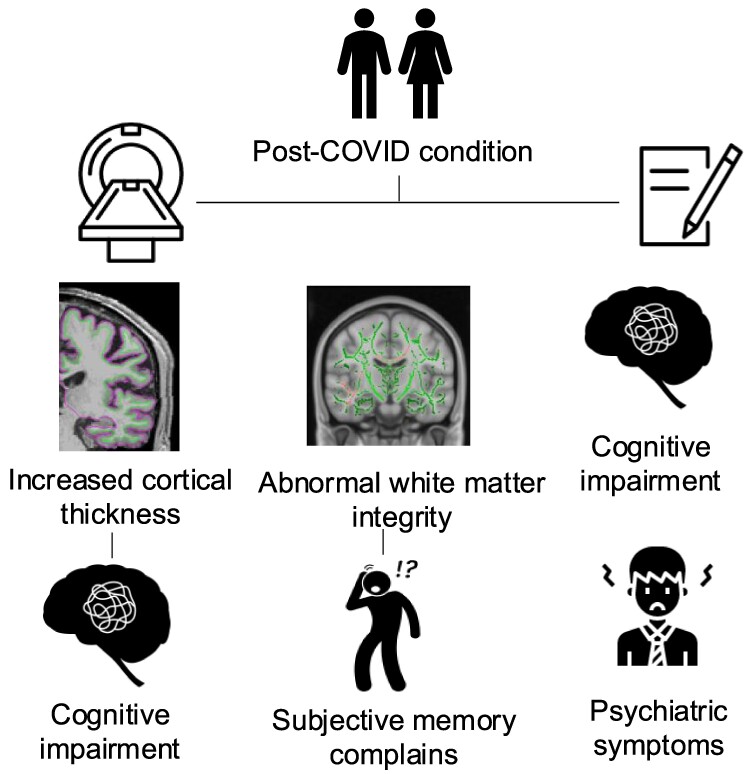

## Introduction

The coronavirus disease 2019 (COVID-19) is an infectious disease caused by the severe acute respiratory syndrome coronavirus 2 (SARS-CoV-2). Most people recover fully after the infection, but a substantial number of individuals suffer from diverse multi-systemic symptoms months after.^1^ The post-COVID-19 condition (PCC) is defined as the continuation or development of new symptoms 3 months after the initial infection, with these symptoms lasting for at least 2 months with no other explanation.^2^ PCC is characterized by a substantial diversity of fixed or fluctuating symptoms including fatigue, muscle and body ache, loss of smell and taste and joint pain as the most reported symptoms according to a population-based cross-sectional study including 4722 participants.^3^ The severity and length of these post-COVID manifestations have been related to the severity of the COVID-19 disease, previous comorbidities and female gender.^4^ Nevertheless, presence of PCC is also frequent in mild COVID-19 patients, ranging between 10 and 35%.^5^

The underlying cause of these symptoms remains unclear but evidence suggests that delayed resolution of inflammation, autoimmunity and viral persistence may be overlapping mechanisms that could contribute to the pathogenesis of the disease, probably leading to a dysfunction in the peripheral and CNS.^6^ Hence, individuals with PCC commonly report subjective cognitive complaints especially difficulty concentrating, presence of brain fog and forgetfulness^7^ that have recently been related to elevated serum fibrinogen and D-dimer^8^ as markers of coagulation system function. Respectively, higher D-dimer levels have been related to objective cognitive deficits, specifically poorer delayed verbal recall and psychomotor speed.^9^ Patients also tend to suffer from neuropsychiatric manifestations, such as depression, anxiety and sleep disturbance,^10,11^ that exert a significant impact on their functional autonomy. Moreover, objective neuropsychological assessments corroborate self-reported symptoms showing impairments in attention,^12^ executive functions,^13-15^ memory,^14,15^ processing speed^13,15^ and language.^13^ The presence of cognitive impairment in patients with post-COVID has been related to COVID-19 severity, describing poorer performance in those individuals who were hospitalized during the acute SARS-CoV-2 infection.^15^ However, some studies have found significant cognitive deficits regardless of the COVID-19 severity.^12^ Comprehension of the underlying pathological mechanisms could be essential to palliate these symptoms related to the functioning of the CNS.

Studies are also an evidence for structural brain changes after COVID-19 infection. First findings were based on CT and MRI. In a review and meta-analysis of structural neuroimaging findings including 1394 COVID-19 patients from 17 studies using clinical MRI or CT, the most common findings were olfactory bulb and white matter (WM) abnormalities, followed by acute/subacute ischaemic infarction and encephalopathy.^16^ Nevertheless, these findings were mainly based on acute stage of the disease.

In 2022, a longitudinal MRI work performed a region of interest-based analyses in a large sample of 394 infected participants from the UK Biobank, and found reductions of cortical thickness (CTh) in memory and olfactory-related brain.^16^ Recently Petersen *et al*.,^17^ reported slightly higher mean cortical CTh in individuals recovered from SARS-CoV-2 infection compared to healthy controls (HC), but differences were not statistically significant. Beyond studies including infected individuals, as the ones mentioned above, there is a growing interest in understanding the long-term sequelae in actual PCC. Nevertheless, evidence related to structural brain abnormalities in this condition is scarce and controversial. In this regard, two recent studies reported opposite results, whereas Serrano del Pueblo *et al*.,^18^ found thinner CTh in long-COVID participants with neurological symptoms in the left temporal gyrus, compared to infected–recovered controls, Besteher *et al*.,^19^ reported higher CTh in patients with long-COVID in extended cortical regions, compared to HC. Disparities are also found in studies evaluating grey matter (GM) volume. While a cross-sectional study found larger GM volume in participants long-COVID suffering from neuropsychiatric symptoms in fronto-temporal areas, insula, hippocampus, amygdala, basal ganglia and thalamus in both hemispheres, compared to HC using a voxel-based morphometry (VBM) approach;^20^ another study found decreased volumes of the left thalamus, putamen and pallidum in individuals suffering post-COVID fatigue using a region of interest-based subcortical analysis.^21^ Even some studies have not found significant differences in GM volume between patients with long-COVID and subjective cognitive complains and controls using a VBM approach.^22^ Differences in sample recruitment and the use of diverse methodologies or differences in evolution time from infection may be causing the discrepancies when studying structural brain changes in this condition, hindering the reaching of agreement on them.

After initial studies reporting WM hyperintensities in individuals infected with COVID-19,^23,24^ diffusion-weighted MRI has also been used to study microstructural WM changes in individuals with previous COVID-19 infection. These studies reported lower fractional anisotropy (FA),^25^ higher mean diffusivity (MD),^1^ higher axial diffusivity (AD)^26^ and lower radial diffusivity (RD)^25^ compared to controls. Notwithstanding, just a few studies have explored these microstructural abnormalities in individuals with post-COVID condition and reported discrepant findings, whereas Serrano del Pueblo *et al*.,^18^ found lower FA and higher RD in patients with long-COVID, compared to infection-recovered controls, Díez-Cirarda *et al*.,^22^ found lower MD and AD values in these patients, compared with HC.

There is a growing interest in elucidating the possible relationship between these structural brain changes and cognition in PCC. Indeed, several studies have already reported association between neuroimaging measures and cognitive performance including a positive correlation between short-term memory scores and thalamic volume,^21^ between overall cognition, verbal fluency, memory and attention and FA,^18^ and between CTh and memory performance.^27^

In this regard, the identification of possible structural brain abnormalities in patients with post-COVID and with and without cognitive impairment based on clinically meaningful criteria is crucially relevant. Only two previous studies reported significant results based on this approach, while Serrano *et al*.,^18^ found no cortical changes between the subgroups of patients classified by degree of overall cognitive impairment based on the standardized NEURONORMA for Spanish population, Besteher *et al*.,^19^ classified patients with long-COVID and with [Montreal Cognitive Assessment (MoCA) < 26] or without (MoCA ≥ 26) cognitive deficits and showed cortical changes in both groups compared to non-infected controls, but did not find differences between the PCC subgroups. In this last study, the authors showed an increase in CTh across different PCC groups compared with non-infected healthy individuals and suggested a progression with more increased thickness in patients with long-COVID exhibiting significant cognitive deficits (MoCA < 26), followed by patients with long-COVID without cognitive deficits (MoCA ≥ 26) and finally, recovered COVID-19 survivors.

Our study aimed to investigate structural brain integrity using a multimodal MRI approach and explore the correlation with patient's cognitive dysfunction, trying to explain the possible brain changes underlying the persistence of cognitive symptoms in patients suffering from post-COVID condition. Characterizing this condition, especially PCC individuals with cognitive impairment, is of crucial interest as they are a potentially at-risk population for age-related diseases that should be monitored over time.

## Materials and methods

### Participants

The sample comprised 165 participants from the Nautilus Project (ClinicalTrials.gov IDs: NCT05307549 and NCT05307575), 128 with post-COVID condition and 37 HC. The sample partially overlaps with previous studies.^15,28,29^ The inclusion criteria for the PCC group were as follows: (i) age between 18 and 65 years; (ii) confirmed diagnosis of COVID-19 according to WHO criteria and (iii) at least 12 weeks after the infection. The exclusion criteria were: (i) established diagnoses before COVID-19 disease of neurological, psychiatric, neurodevelopmental disorder, systemic pathologies known to cause cognitive deficits and (ii) motor or sensory alterations that obstruct the neuropsychological evaluation. Thirty-one PCC participants were vaccinated before (24.20%) and 69 after (53.90%) Sars-CoV-2 infection and 18 were not vaccinated (14.10%) (Supplementary Table 1). Participants in the HC did not have a history of symptoms compatible with SARS-CoV-2 infection nor a positive test prior to the study. The same exclusion criteria for the PCC group were applied to the HC group. All participants were Spanish native speakers.

The recruitment was performed between June 2021 and December 2023, which comprehends different waves of COVID-19 infection. Supplementary Figure 1 shows average weekly mutations counts in Spain and Catalonia during this period (enabled by Data from GISAID https://www.gisaid.org/). The study was conducted with the approval of the Drug Research Ethics Committee (CEIm) of Consorci Sanitaria de Terrassa (CEIm code: 02-20-107-070) and the Ethics Committee of the University of Barcelona (IRB00003099). All participants provided written informed consent after full explanation of the procedures.

### Neuropsychological assessment

This study comprised two sessions, the detailed evaluation was described elsewhere.^29^ In the first session, questionnaires were administered to the participants to gather information about demographic factors, data on COVID-19 infection and previous comorbidities. Age, sex, years of education, ethnicity, citizenship, profession and income were registered. Participants were also asked about their medical history and behaviour related to their health. Their COVID-19 experience (symptoms, treatment, hospitalization and time since diagnosis) and information on their post-COVID symptoms (including cognitive ones) were also questioned.

In the second session, each participant underwent a cognitive assessment with a comprehensive neuropsychological battery. The Montreal Cognitive Assessment (MoCA) was used as a general cognitive screening tool.^30,31^ To assess abstract reasoning, the Matrix subtest from the Wechsler Adult Intelligence Scale III was used.^32^ Verbal learning (total learning) and memory (delayed recall) were evaluated using the Spanish version of Rey's auditory verbal learning test (RAVLT);^33,34^ whereas the immediate and the 30-min delayed recall test from the Rey–Osterrieth Complex Figure Test^35^ was used for visual memory. The Wechsler Adult Intelligence Scale III Digit Span subtest was used to measure working memory (digit span backwards) along with verbal attention (digit span forward).^32^ Parts A and B of the Trail Making Test were administered to measure visual scanning, motor speed and attention and mental flexibility.^36^ Related to semantic fluency, it was assessed using the category ‘animals’^37^ by considering the number of animals recalled in 1 min. Also, the number of words beginning with P, M and R recalled in 1 min each was registered to measure phonemic fluency. The Stroop test consists of three subtests: words, colours and colour words that conflict with the colour in which they are presented.^38^ Visual scanning, tracking and motor speed were assessed by the digit symbol test from the Wechsler Adult Intelligence Scale III.^32^ The Boston naming test was used to evaluate language.^39^

The neuropsychological test scores were validated, with normative data available for our country, adjusted by age and education levels. Direct scores from these tests were transformed into *z*-scores based on normative scales recommended in previous literature, including the Digit Symbol, Matrix, Digit Span,^32^ RAVLT,^33^ Trail Making Test, Stroop, phonemic fluency and semantic fluency tests, Boston naming test and Rey–Osterrieth Complex Figure Test.^40^

Lower *z*-scores reflect lower cognitive performance. PCC participants were grouped into 2 categories based on the *z*-scores obtained for each cognitive test. Participants were classified as ‘not altered’ if they presented non or one cognitive test with *z*-score ≤ −1.5 or ‘altered’ if they presented more than one cognitive test with *z*-score ≤ −1.5.

Emotion recognition was evaluated with the reading the mind in the eye test.^41^ Moreover, the world accentuation test (TAP) was also included as an estimate of premorbid IQ^42^ and Spanish version of the smell identification test—40 items (UPSIT-40)^43^ was used to measure olfactory function. In addition to cognitive measures, we used the Chalder fatigue scale^44^ to assess fatigue, the generalized anxiety disorder 7-item scale^45^ to assess anxiety, the Patient Health Questionnaire-9^46^ to assess depression and memory failures of everyday test^47^ to assess subjective memory complains. All evaluations were performed by trained neuropsychologists. The interval between the infection and the evaluation sessions was also registered.

### MRI acquisition

A 3T scanner (MAGNETOM Prisma, Siemens, Germany) was used to acquire MRI data. The protocol for scanning encompassed high-resolution 3D T1-weighted images captured in the sagittal plane [repetition time (TR) = 2400 ms, time to echo (TE) = 2.22 ms, inversion time (TI) = 1000 ms, 208 slices, field of view (FOV) = 256 mm, 0.8 mm isotropic voxel], two diffusion-weighted imaging acquisitions with equal parameters (TR = 3230 ms, TE = 89.20 ms, voxel size = 1.5 mm^3^, 99 diffusion directions at *b* = 0, 1500 and 3000 s/mm^2^, flip angle 78°, 92 slices, FOV = 210 mm; slice thickness 1.5 mm) but flipped phase-encoding direction (anterior–posterior and posterior–anterior), and an axial FLAIR sequence (TR = 6000 ms, TE = 397 ms).

Later pre-processing and analyses were accomplished at the Neuroimaging Laboratory of the Medical Psychology Unit, Department of Medicine, University of Barcelona, Spain.

### CTh and volumetric measures

FreeSurfer software (FS, version 7.1; available at: https://surfer.nmr.mgh.harvard.edu/) was used to pre-process structural MRI and determine CTh. The 3D cortical surface model applied for this estimation was generated using intensity and continuity information, as detailed by the authors.^48,49^ Results for each subject were carefully examined through visual inspection to provide precision of registration, skull stripping, segmentation and cortical surface reconstruction. A circularly symmetric Gaussian kernel across the surface with a full width at half maximum of 15 mm was used to smooth CTh maps.

The FS pipeline was also used to obtain summary CTh values among cortical parcellations and mean Cth of both hemispheres. To obtain a mean value of the CTh of the whole brain, we used the following expression:


$$\mathrm{Bh}\_\mathrm{thickness}=\frac{\left(\left(\mathrm{lh}\;\mathrm{thickness}\cdot \mathrm{lh}\;\mathrm{surfarea}\right)+\left(\mathrm{rh}\;\mathrm{thickness}\cdot \mathrm{rh}\;\mathrm{surfarea}\right)\right)}{\left(\mathrm{lh}\;\mathrm{surfarea}+\mathrm{rh}\;\mathrm{surfarea}\right)}.$$

Intergroup CTh comparisons were assessed applying a vertex-by-vertex general linear model (GLM) with FS, Monte Carlo Null-Z Simulation with 10 000 iterations was applied to CTh maps to provide cluster-wise correction for multiple comparisons; the cluster-defining threshold was set at 1.3, in both directions (abs). Results were set with a threshold at a corrected *P*-value of 0.05 and visualized with Freeview from FS.^50^ Then vertex-wise Cohen's *d* effect size was estimated for CTh. Only effect sizes *d* > 0.3 were considered.

Automated subcortical segmentation performed with FS was used to estimate subcortical volumetry.^51^ In order to correct volumetric data for intracranial inter-individual differences, estimated total intracranial volume was obtained.

### GM volume and WM hyperintensities

T1-weighted structural images were segmented by means of SPM's toolbox CAT12 (https://neuro-jena.github.io/cat/) using the standard pipeline. We also requested the segmentation toolbox to provide the maps of WM hyperintensities, which were lately quantified with the GET-TIV tool. We constructed the template of the sample and normalized it to Montreal Neurological Institute (MNI) space using DARTEL.^52^ The resulting warps (DARTEL flow-fields + affine transformation of sample's template to MNI) were applied to original GM segments. Normalized GM images were multiplied by the Jacobians of the warp from native space to MNI to preserve the volume of original data in the MNI space (modulation). Spatially normalized and modulated GM maps where posteriorly smoothed with an 8 mm-full width at half maximum Gaussian Kernel.

### Diffusion tensor imaging

Pre-processing of diffusion MRI images included correction for Eddy current distortions and subject's motion. Following the pre-processing, we employed the diffusion tensor model fit function within FMRIB software library (FSL) to fit the diffusion tensor model to each voxel. The set of images for each subject included 14 non-diffusion-weighted with a *b*-value of 1500 s/mm^2^, and 94 diffusion-weighted volumes at *b* = 1500 s/mm^2^, in an anterior–posterior–posterior–anterior acquisition. Using a diffusion tensor model fit, individual FA maps were obtained. A statistical analysis at voxel level of the FA, MD, RD and AD was carried out with the tract-based spatial statistics.^53^ Tract-based spatial statistics performs non-linear registration [using non-linear image registration tool (FMRIB)] of FA images from diffusion tensor model fit to the MNI standard space, creating a mean FA skeleton that represents the central structure of all WM tracts shared by the entire group. Each subject's FA image was mapped onto the skeleton and the resulting FA skeleton images were fed into a GLM to compare the two patient groups and identify vertex-wise differences in FA skeleton maps. The same procedure was employed to obtain the MD, RD and AD maps. FSL's randomise^54^ was used to compute group analysis. Results were visualized with Fslview from FSL (https://zenodo.org/records/11047709).

### Blood biomarkers

A subsample of PCC participants [38 non-hospitalized, 11 hospitalized and 17 hospitalized intensive care unit (ICU) admitted] also underwent blood extraction during the second session. Levels of blood biomarkers: interleukin 6, reactive protein C, nerve growing factor, ferritin, thrombomodulin, D-dimer, endothelin 1, glial fibrillary acidic protein and malondialdehyde were measured. Interleukin 6, nerve growing factor and reactive protein C were analysed by Bio-Plex [Bio-Plex^®^ Handheld magnetic masher (Bio-Rad catalog # 171020100)], samples were diluted and analysed in duplicate when needed, assays were performed according to manufacturer's protocol and the plate was read in 800TS microplate reader (BioTek) using the instrument settings in manufacturer's protocol. Concentration results were calculated from standard curve. For D-dimer, glial fibrillary acidic protein, thrombomodulin, ferritin, endothelin-1 and malondialdehyde levels in blood serum, we used the following enzyme-linked immunosorbent assay (ELISA): Human Thrombomodulin ELISA Kit (CD141) (Merck Cat. #RAB0648-1KT), Human GFAP ELISA Kit (Elabscience Cat. #E-EL-H6093-96T), Human Thrombomodulin ELISA Kit (CD141) (Abcam Cat. #ab46508), Human Ferritin ELISA Kit (Merck Cat. #RAB0197-1KT), Endothelin 1 ELISA Kit (Abcam Cat. #ab133030) and malondialdehyde. Samples were diluted and analysed in duplicate when needed and standards were prepared according to manufacturer's protocol and read it at 450 nm. Concentration results were calculated from standard curve.

### Statistical analysis

Statistical analyses of demographic, neuropsychological, blood biomarkers levels and volumetric data were carried out using the statistical package SPSS-27.0.1 (2020; Armonk, NY, USA: IBM Corp.) and Matlab (The MathWorks Inc., 2022). Group differences in demographic and clinical data were analysed using Student's *t*, ANOVA or non-parametric analysis (Kruskal–Wallis H and Mann–Whitney U) when needed. Pearsons's χ^2^ test was carried out for categorical variables. A GLM, non-parametric F testing with 1000 permutation was used to determine differences in cognitive performance, neuropsychiatric symptomology, subcortical volumetry, whole-brain cortical mean thickness and whole-brain mean diffusion parameters (FA, MD, RD and AD) among groups; *P*-values were then corrected for false discovery rate (FDR). Correlations between mean CTh and mean diffusion parameters with cognitive raw scores and other variables of interest were carried out using Pearson's or Spearman's correlation when needed and adjusted for age and sex. For all analyses, significance threshold was set at a corrected level of *P* < 0.05. Plots were designed with R studio (https://www.R-project.org) using the ggplot2 package (https://ggplot2.tidyverse.org).

## Results

### Sociodemographic and clinical characteristics

Participants’ sociodemographic characteristics are shown in Table 1. PCC participants were similar to controls in age, education, sex and estimated IQ. Forty-one (32%) PCC patients were hospitalized during the acute infection of SARS-CoV-2, of which 24 (19%) were admitted to the ICU. Among the reported comorbidities, diabetes mellitus was more prevalent among PCC participants (Supplementary Table 2).

**Table 1:** Demographic and clinical characteristics of PCC participants and HC

	HC (*n* = 37)	PCC (*n* = 128)	Test stat/*P*-value
Demographic characteristics			
Age in years, mdn (min–max)	54.00 (41–61)	52.00 (30–65)	−0.88/0.377
Education in years, mdn (min–max)	16.00 (9–21)	15.00 (8–24)	−0.66/0.511
Sex, female, *n* (%)	25 (67.6)	98 (76.6)	1.22/0.269
IQ, mdn (min–max)	107.00 (98–114)	106.00 (85–116)	−1.29/0.197
Vaccine, yes, *n* (%)	26 (70.27)	100 (84.80)	4.14/0.042*
Hospitalization, *n* (%)		41 (32.0)	
ICU,^b^ *n* (%)		24 (18.8)	
Interval time COVID-MRI in months, mdn (min–max)		18.00 (3–44)	
Interval time NP-MRI in months, mdn (min–max)	0.00 (0–3)	0.00 (0–5)	−0.60/0.546
Clinical data^a^			
PHQ-9, mdn (min–max)	3.11 (0–9)	10.00 (0–25)	29.41/0.001**
GAD-7, mdn (min–max)	3.00 (0–13)	5.00 (0–21)	10.47/0.003**
MFE, mdn (min–max)	7.00 (1–22)	23.00 (0–51)	29.21/0.001**
CFQ, mdn (min–max)	1.50 (0–10)	10.00 (0–11)	52.85/0.001**

CFQ, Chalder fatigue scale; GAD-7, generalized anxiety disorder 7-item scale; HC, healthy controls; ICU, intensive care unit; IQ, intelligence quotient; max, maximum; mdn, median; min, minimum; MFE, memory failures of everyday; min, minimum; MRI, magnetic resonance imaging; NP, neuropsychological assessment; PCC, post-COVID condition; PHQ-9, Patient Health Questionnaire-9. Group differences were tested using independent U de Mann–Whitney or GLM. Differences in categorical variables were analysed with Pearson's χ^2^ test. *P-value < 0.05. **P-value < 0.05 FDR-corrected. ^a^Due to missing data, PHQ-9 and GAD-7 *n* = 138; MFE and CFQ *n* = 139 in PCC group. For control group, all four variables *n* = 24. ^b^ICU-admitted participants are included in the total number of hospitalizations.

We obtained data from the neuropsychiatric questionnaires in 115 PCC participants. In this subsample, PCC participants showed higher scores in tests assessing depressive symptomatology, anxiety, memory failures and fatigue compared to HC group, reported as higher scores in Patient Health Questionnaire-9, generalized anxiety disorder 7, memory failures of everyday and Chalder fatigue scale questionnaires (Table 1).

Post-COVID symptoms were also evaluated. Fatigue (74%), shortness of breath (60%) and pain (55%) were the most frequent reported symptoms, as shown in Supplementary Fig. 2.

A blood extraction was acquired for a subsample of 66 PCC and blood biomarkers were quantified (Supplementary Table 3).

### Neuropsychological assessment

Table 2 shows differences in cognitive performance between PCC and HC groups. PCC showed statistically significant poorer performance compared to control group in MoCA, RAVLT total score, RAVLT delayed recall, RAVLT recognition, digit span forward, digit span backward, digit symbol coding, Trail Making Test part A, Stroop words, Stroop colors, but not Stroop interference, PMR and semantic (animals) fluencies, and UPSIT. All differences survived significance after FDR correction. The intergroup comparisons showed medium to large effect sizes. No differences between groups were found in Matrix, Rey–Osterrieth Complex Figure Test immediate and delayed memory accuracy, Trail Making Test part B, Stroop interference, Boston naming test or reading the mind in the eyes test.

**Table 2:** Differences in neuropsychological assessment of PCC participants compared to HC group

	HC		PCC			
	*n*	Mdn (min–max)	*n*	Mdn (min–max)	*F*/*P*-value	Cohen's *d*
MoCA	26	28.00 (25–30)	120	27.00 (18–30)	5.70/0.032**	0.51
Matrix	26	20.00 (5–26)	128	19.00 (5–50)	1.12/0.293	
RAVLT total	37	50.00 (35–65)	128	45.50 (23–65)	8.35/0.019**	0.53
RAVLT delayed recall	37	10.00 (4–15)	128	9.00 (1–15)	7.34/0.019**	0.50
RAVLT recognition	37	14.00 (10–15)	127	13.00 (3–15)	7.24/0.019**	0.49
ROCF immediate memory accuracy	26	20.50 (8.5–30)	88	19.00 (1–30)	1.88/0.208	
ROCF delayed memory accuracy	26	21.00 (8–29)	88	18.50 (1–30)	1.94/0.188	
Digit span forward	26	6.00 (4–8)	128	6.00 (3–9)	8.15/0.019**	0.62
Digit span backward	26	5.00 (4–8)	128	4.00 (2–8)	11.41/0.011**	0.73
DSC coding	26	77.00 (37–100)	128	66.00 (21–100)	12.36/0.011**	0.76
TMTA	37	27.00 (15–76)	128	35.00 (13–180)	6.02/0.031**	−0.46
TMTB	37	66.00 (31–170)	128	72.00 (10–300)	3.80/0.080	
Stroop W	35	106.00 (62–131)	125	95.00 (20–144)	12.26/0.011**	0.70
Stroop C	35	70.00 (46–92)	125	61.00 (21–108)	10.84/0.011**	0.62
Stroop WC	35	42.00 (12–65)	125	38.00 (13–96)	3.77/0.070	
Phonemic fluency (PMR)	26	48.50 (23–77)	127	39.00 (14–83)	20.11/0.011**	0.97
Semantic fluency (animals)	37	24.00 (11–35)	128	20.00 (8–38)	8.67/0.013**	0.57
BNT	26	57.00 (47–60)	89	54.00 (34–60)	4.47/0.066	
RMET	26	24.00 (15–28)	89	22.00 (15–31)	3.95/0.066	
UPSIT	32	32.00 (27–38)	71	30.00 (11–37)	10.15/0.001**	0.68

BNT, Boston naming test; DSC, digit symbol coding; HC, healthy controls; max, maximum; mdn, median; min, minimum; MoCA, Montreal cognitive assessment; PCC, post-COVID condition; PMR, phonemic fluency; RAVLT, Rey's auditory verbal learning test; RAVLT delayed recall, total recall after 20 min; RAVLT total, sum of correct responses from trial I to trial V; RMET, reading the mind in the eyes test; ROFC, Rey–Osterrieth complex figure test; Stroop W, Stroop words; Stroop C, Stroop colours; Stroop WC, Stroop words-colours; TMTA, trail making test part A; TMTB, trail making test part B; UPSIT, University of Pennsylvania Smell Identification Test. Group differences were tested using GLM. Cohen's *d* effect size is as follows: *d* = 0.2–0.3, small; *d* = 0.5–0.8, medium; *d* = >0.8, high. **P-value <0.05 FDR-corrected.

### Brain alterations and clinical correlations

#### Cortical thickness

**Figure 1:**
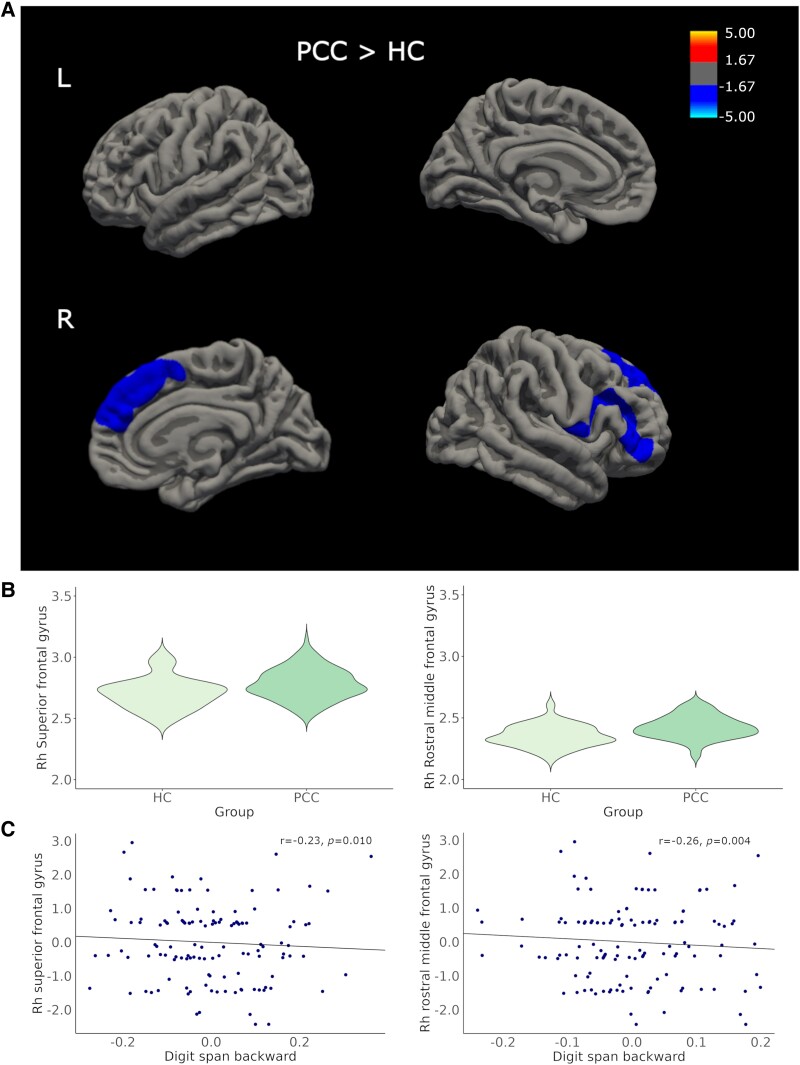
**CTh comparison between PCC and HC.** (**A**) PCC participants showed higher CTh in the right superior frontal (*P* = 0.0008) and the right rostral middle frontal (*P* = 0.0036) gyri. Significant differences are indicated in cool colours. Heat map indicates the average difference in CTh; the scale represents *t*-statistics indicating the magnitude and direction of group differences. Only clusters that survived cluster-extend Monte Carlo corrections for multiple comparisons (*P* < 0.05) are shown. (**B**) Violin plots of mean CTh of significant clusters comparing HC and PCC participants. (**C**) Partial regression plots of mean CTh from between-groups significant clusters and digit span backward raw scores, regressed by age and sex (*r* = −0.23, *P* = 0.010; *r* = −0.26, *P* = 0.004). HC, healthy controls; L, left; PCC, post-COVID condition; R, right; Rh, right hemisphere.

CTh analysis was performed for the 165 participants (37 HC and 128 PCC) in the study, of which 87 were not hospitalized PCC, 17 hospitalized PCC and 24 hospitalized ICU-admitted during the COVID acute infection PCC participants (Table 1). Vertex-wise analyses results showed increased CTh in PCC participants compared to HC group. Clusters with significantly higher CTh were found in the right superior frontal (2572.68 mm^2^; *P* = 0.0008) and the right rostral middle frontal (2234.87 mm^2^; *P* = 0.0036) gyri (Fig. 1A and B and Supplementary Table 4). The intergroup CTh results reached large effect sizes (*d* = 0.8) (Supplementary Fig. 3). No differences were found regarding whole-brain mean CTh between groups [HC median (min–max): 2.43 (2.28–2.59); PCC median (min–max): 2.48 (2.30–2.70). *F*(1.67) (*P* = 0.227)].

We also explored the relation between neuropsychological impairment and CTh from between-groups significant clusters. In the PCC group, we found a significant negative association between digit span backward and the mean CTh from the right middle frontal (*r* = −0.23, *P* = 0.010) and the right superior frontal (*r* = −0.26, *P* = 0.004) gyri (Fig. 1C). Only the correlation between the right rostral middle frontal gyrus remained significant after FDR correction.

None of these correlations were found in the control group.

Time interval between COVID infection and MRI acquisition showed tendency to correlate positively with the mean thickness from the right superior frontal gyrus (*r* = 0.16, *P* = 0.070).

Moreover, no correlation was found between CTh and any blood biomarker.

### GM volume, subcortical volumetric measures and WM hyperintensities

No significant between-group differences were found in GM VBM, subcortical volumetric measures (Supplementary Table 5) or WM hyperintensities in cm^3^ [HC median (min–max): 2.090 (0.62–5.43) PCC median (min–max): 2.091 (0.58–8.25) *F*(0.03), (*P* = 0.874)].

### Diffusion MRI parameters

A subsample of participants underwent the diffusion-weighted imaging acquisitions, of which, 83 were not hospitalized, 10 hospitalized and 5 ICU admitted during the COVID acute infection (Supplementary Fig. 4). In this subsample, the PCC group did not have differences in sociodemographic characteristics compared to HC group in age, education and estimated IQ. However, PCC group showed significant higher proportion of women (88 versus 68%) than HC (Supplementary Table 6). Therefore, sex was introduced as a covariate in all the upcoming analysis between these two groups. Differences in cognition and neuropsychiatric questionnaires between both groups were similar to the ones in the whole sample (Supplementary Tables 7 and 8).

**Figure 2:**
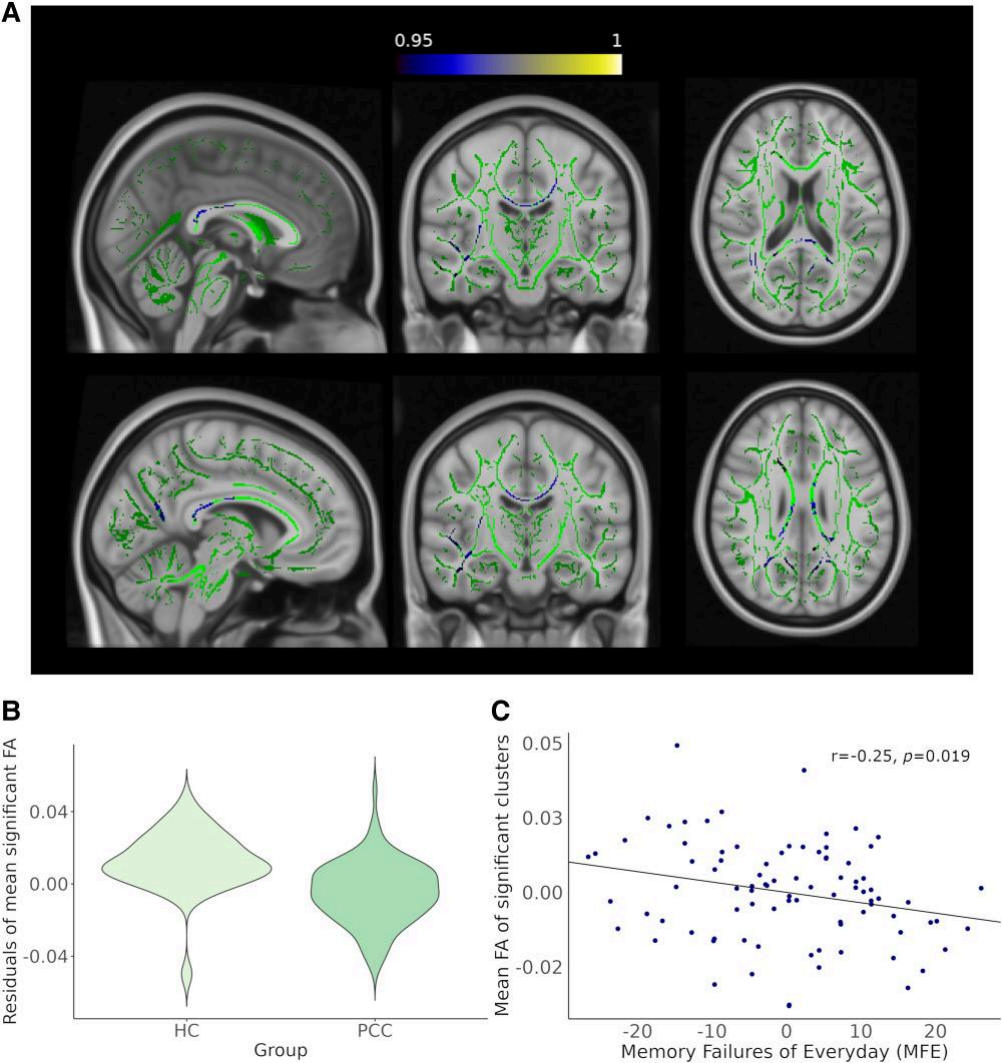
**Tract-based spatial statistics differences between HC and PCC in FA.** (**A**) PCC participants showed lower FA in regions involving the right superior longitudinal fascicles (*P* = 0.04; *P* = 0.048), the right uncinate fasciculus (*P* = 0.049; *P* = 0.049), the forceps major (*P* = 0.049) and the splenium (*P* = 0.038) and genu (*P* = 0.048) of corpus callosum. Significant differences are indicated in cool colours; the scale is represented as alpha (*P*-value = 1 − alpha), meaning 0.95 indicates significant clusters. GLM and FSL's randomize were used to find vertex-wise differences in FA skeleton maps. Results are overlaid on the WM skeleton (green) and displayed over sections of the MNI 152 standard brain at *P* < 0.05 FWE corrected. (**B**) Violin plots of the residuals of mean FA when controlled by sex, comparing PCC and HC groups. (**C**) Partial regression plots of mean FA extracted from between-groups significant clusters and MFE score, regressed by age and sex (*r* = −0.25, *P* = 0.019). FA, fractional anisotropy; FWE, family-wise error; HC, healthy controls; MFE, memory failures of everyday; PCC, post-COVID condition.

Results of diffusion tensor imaging (DTI) analyses showed lower FA in PCC participants compared to HC group. Significant clusters were mainly located in the right superior longitudinal fasciculus, the splenium and genu of the corpus callosum, the right uncinate fasciculus and the forceps major (Fig. 2A and B and Supplementary Table 9). Moreover, significant lower values of whole-brain mean FA were found in PCC group compared to HC (*P* = 0.016); however, this difference did not survive FDR correction. No differences between groups were found in whole-brain mean MD, RD or AD (Supplementary Table 10).

No correlation was found between FA and neuropsychological values neither with any blood biomarker. Nevertheless, we found a significant negative association between subjective memory failures evaluated by memory failures of everyday questionnaire and mean FA from significant clusters (*r* = −0.25, *P* = 0.019, FDR-corrected) in PCC group (Fig. 2C). No correlations were found in the control group.

### Clinical cognitively altered versus non-cognitively altered patients

Seventy-one PCC individuals were classified as cognitively altered and 57 as non-altered PCC patients. Cognitively altered group included 46 non-hospitalized, 10 hospitalized and 15 hospitalized ICU-admitted PCC participants; and non-cognitively altered group included 41 hospitalized, 7 hospitalized and 9 hospitalized ICU-admitted PCC participants. Differences between both PCC groups were found in age, education and IQ (Supplementary Table 11). Supplementary Fig. 5 describes mean *z*-scores in every test among groups. Significant differences between altered and non-altered PCC were found in all tests. Moreover, Supplementary Fig. 6 shows the frequency of *z*-scores ≤−1.5 in each neuropsychological test. Of note that higher frequencies are present in all cognitive tests in PCC participants classified as altered, compared with PCC participants classified as not altered. Differences in psychiatric questionnaires between groups are shown in Supplementary Table 12.

When exploring differences in blood biomarkers between PCC groups stratified according to cognitive impairment, higher levels of interleukin 6 and lower levels of D-dimer were found in cognitively altered PCC participants, compared with non-cognitively altered PCC. No differences were found between groups after FDR correction (Supplementary Table 13).

**Figure 3:**
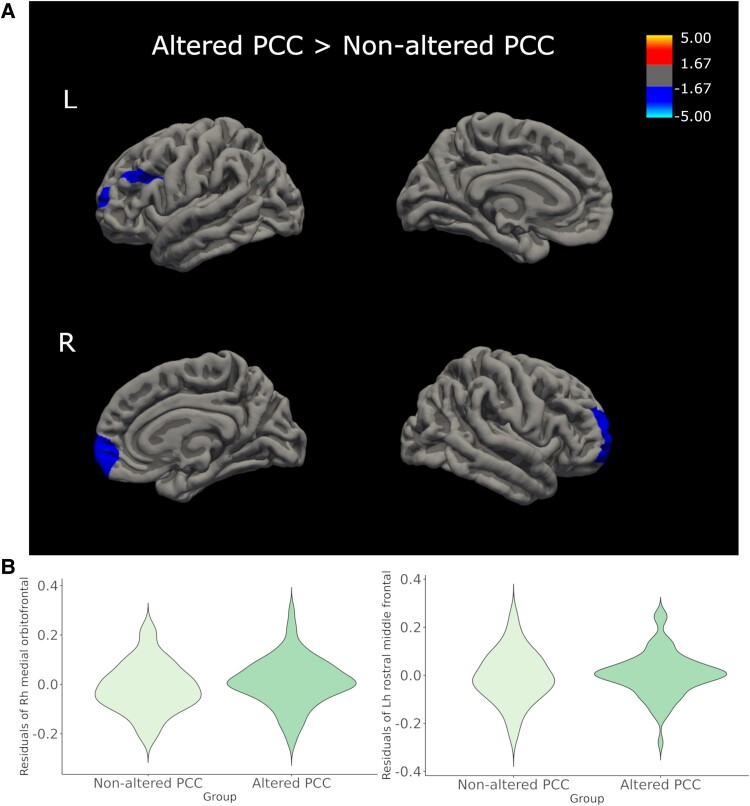
**Group comparison of CTh between cognitively altered and non-cognitive altered PCC participants.** (**A**) Altered PCC participants showed higher CTh in right medial orbitofrontal and left rostral middle frontal. Significant differences are indicated in cool colours. Only clusters that survived cluster-extend Monte Carlo corrections for multiple comparisons (*P* < 0.05) are shown. (**B**) Violin plots of the residuals of mean CTh of significant clusters comparing altered and non-altered PCC participants, adjusted for age and education. L, left; Lh, left hemisphere; PCC, post-COVID condition; R, right; Rh, right hemisphere.

Regarding structural changes, we found two clusters with increased CTh in PCC participants with altered cognition compared with PCC participants without altered cognition, adjusting for age and education (Fig. 3 and Supplementary Table 14). Clusters with significant higher CTh were found in the right medial orbitofrontal (*P* = 0.0036) and the left rostral middle frontal (*P* = 0.0437) gyri, similar to the differences found between PCC participants and HC.

Related to microstructural WM changes, no differences were found when comparing PCC participants with and without altered cognition, adjusting for age and education. No significant differences were found between groups regarding GM VBM, volumetric subcortical measures or WM hyperintensities neither.

### Role of hospitalization during the acute infection

Additionally, we aimed to investigate the possible effect of severity during the acute infection of SARS-CoV-2. Differences in sociodemographic characteristics between PCC classified according to hospital admission during acute infection are shown in Supplementary Table 15.

When analysing structural brain differences splitting the sample into 4 groups: hospitalized-ICU, hospitalized PCC, non-hospitalized PCC and HC participants, non-hospitalized PCC participants showed higher CTh in the right parsopercularis [*F*(−3,26), *P* = 0.00020, 2702.81 mm^2^, MNIX = 48.5, MNIY = 9.0, MNIZ = 12.4] compared to controls, adjusting for sex. Differences were also found between non-hospitalized and hospitalized PCC, with higher CTh present in non-hospitalized PCC participants in the right superior temporal [*F*(2.48), *P* = 0.01851, 1701.44 mm^2^, MNIX = 49.7, MNIY = −27.4, MNIZ = −1.0]. No differences were found between hospitalized PCC and HC group or hospitalized-ICU PCC and HC group, nor between hospitalized and hospitalized-ICU PCC or non-hospitalized PCC participants and hospitalized-ICU PCC participants.

Results remained significant when CTh analysis was performed between 3 groups: HC, hospitalized and non-hospitalized PCC participants, including hospitalized-ICU participants in the hospitalized PCC group (Supplementary Table 16). Non-hospitalized PCC participants also showed higher CTh compared to HC [*F*(−3.35), *P* = 0.00020, 2628.88 mm^2^, MNIX = 35.9, MNIY = 33.1, MNIZ = 14.8], but differences between hospitalized and non-hospitalized PCC disappeared after adjusting for time interval between COVID infection and MRI.

Regarding DTI analysis, sociodemographic characteristics of the analysed subsample are shown in Supplementary Table 17. Non-hospitalized PCC participants showed lower FA values in the splenium of corpus callosum (*P* = 0.043, 391 voxels, MAX-X = −9, MAX-Y = −34, MAX-Z = 22), compared to HC. No differences were found between hospitalized and HC group neither between hospitalized and non-hospitalized PCC groups in FA values. Unfortunately, due to the limited sample size (*n* = 5) of the hospitalized-ICU PCC group, between-group comparison analyses including this subgroup were not performed.

No significant differences were found between groups regarding GM VBM or volumetric subcortical measures. However, significant differences were found between hospitalized and non-hospitalized PCC participants in white matter hyperintensities (WMH) [non-hospitalized PCC median (min–max): 1.51 (0.58–5.13) and hospitalized PCC median (min–max): 2.19 (0.99–8.06), *F*(10.34), *P* = 0.003)]. No other differences were found between groups in WMH.

## Discussion

This study provides further evidence that support the hypothesis that structural brain alterations are associated with neuropsychological performance in PCC, analysing differences between PCC patients with and without clinically meaningful cognitive impairment. Our results evidenced cortical thickening and decreased microstructural WM integrity in PCC, as well as low performance in working memory, verbal memory, mental processing speed, verbal fluency and executive functions. CTh correlated with working memory performance and was identified only in those PCC patients with altered cognition, whereas abnormal microstructural WM integrity was associated with PCC and related with subjective memory but not with objective neuropsychological performance or other clinical variables.

In our study, individuals with post-COVID condition showed lower performance in cognitive domains involving working and verbal memory, processing speed, verbal fluency and executive functions; compared to HC. As expected, the results concord with results from Ariza *et al*.,^15^ using a larger sample that partially overlapped participants of this study. Closely, previous studies have reported memory, attention, executive functions, processing speed and concentration as the most affected cognitive domains in individuals with post-COVID condition.^13-15^ In this regard, our results showed medium-to-large effects, as indicated by effect size calculations, and half of the sample were considered cognitively impaired based on clinical criteria. Moreover, cognitively altered PCC performed significantly worse in all cognitive domains, compared with non-altered PCC participants. This is relevant considering that the studied sample is relatively young (under 65 years of age) and highlights the importance of monitoring these individuals over time due to the possible evolution to age-related diseases.

Regarding the neuroimaging findings, cortical thickening in PCC patients mainly involving the right frontal lobe was found. However, when observing the whole-brain effect sizes, this cortical thickening seemed to be more extensive and bilateral. Similar to our results, Besteher *et al*.,^19^ studied a sample of 61 PCC patients and found higher CTh PCC patients compared to non-infected HC but also to controls with prior infection. Petersen *et al*.,^17^ also showed slightly higher CTh in COVID-19 patients without PCC compared to the HC group, even though differences were not significant after statistical correction. This cortical thickening present in patients with post-COVID may have its origin in a neuroinflammation process caused by the virus entrance in the CNS. In fact, several studies have proved that SARS-CoV-2 spike S1 subunit can induce by itself a neuroinflammatory response and microglia activation.^55,56^ Moreover, Besteher *et al*.,^19^ reported increased levels of IL-10, IFNγ and sTREM2 in serum related to cortical thickening in patients suffering from post-COVID condition. These results suggest a possible underlying inflammatory process explaining cortical alterations in patients with post-COVID. Interestingly, cognitive impairment present in patients with long-COVID has also been associated with the presence of inflammation and blood brain barrier disruption,^57^ indicating inflammation could be the cause of brain changes in these patients, that will finally lead to cognitive impairment. Nevertheless, our study did not find relationship between cortical thickening and inflammation biomarkers, possibly because serum levels of biomarkers tend to normalize over time, hindering their detection months after the viral infection, as other authors have already proposed.^58^ Importantly, we could not provide cognitive and imaging data at the time of the acute infection, which prevents us from understanding the evolution of this probable neuroinflammation and the distinction between acute and more chronic inflammatory and structural brain changes. Longitudinal studies with data collection during the acute infection or the addition of an infected control group would help to clarify this question.

Our results showing increased CTh are not in line with the longitudinal study by Douaud *et al*.,^59^ which reported cortical thinning in the parahippocampal gyrus and the lateral orbitofrontal cortex. Even though this study has been widely cited and referenced by other authors, their participants are not PCC but individuals recovered from COVID-19 infection and their study design is broadly different to ours, which difficult the direct comparison with our study. Another study reporting lower CTh in PCC patients compared with individuals recovered from COVID-19 infection.^18^ This mentioned study used a 1.5T scanner to study a smaller sample of PCC participants with neurological symptoms and shorter time since infection in comparison to our participants. Notably, contrary to our results, the authors did not find correlations between cortical thinning and cognition or clinical symptoms. Indeed, we found an association between impaired working memory and cortical thickening in right frontal brain regions. To our knowledge, only one recent study reported correlation between CTh and performance in memory domain.^27^ However, this study was limited by a small sample of individuals with post-COVID condition, it did not include a control group and used a multiple regression analyses approach based on mean thickness region of interest data, without multiple comparison correction. Overall, the methodological differences and the inclusion of intracranial volume as covariate in the CTh regression analyses could explain the discrepancies between studies.

Moreover, we also found a tendency of CTh to correlate positively with time since infection, potentially indicating patients with a higher time between infection and assessment may have worse structural changes as they have been carrying this PCC longer time with no recovery. Servier *et al*.,^60^ followed 2197 patients with post-COVID and found that 91% of these patients recovered slowly, but a 4% had persistent condition even after 2 years of symptoms onset. In our sample, the mean interval since infection is 20 months; our study includes 47 participants with persistent symptomatology after 2 years of infection onset.

Regarding GM abnormalities, we did not find changes in GM volume or subcortical volumetry between-groups, even though some previous studies do report changes in these values in PCC individuals.^20-22^ Díez-Cirarda *et al*.,^22^ found a correlation between GM volume and cognitive performance. Nevertheless, associations in this study were not controlled for age and sex even though did survive multiple comparison correction.

The current study also found widespread microstructural WM changes. A FA decrease, indicating a loss of microstructural integrity, was found in bilateral regions mainly involving the right hemisphere in PCC patients. Comparably to our results, Díez-Cirarda *et al*.,^22^ found WM changes in patients with post-COVID, observing decreased MD and AD in a voxel-wise approach and decreased whole-brain mean FA in patients with post-COVID. Authors additionally found correlations between mean MD and mean AD with verbal and visual memory. In this line, Serrano del Pueblo *et al*.,^18^ found lower values of FA and RD in patients with post-COVID in several WM areas of both hemispheres including the cingulum bundle, the rostrum, genu and splenium of the corpus callosum, the uncinate fasciculus, the superior and inferior longitudinal fasciculus, parts of the arcuate fasciculus and medial and lateral occipitotemporal WM. Interestingly, we also found lower values of FA in the superior longitudinal fasciculus, the corpus callosum and the uncinated fasciculus, WM tracts that are relevant for higher order processing. However, whereas authors reported a relationship between lower FA and episodic memory, overall cognitive function, attention and verbal fluency scores, we only found negative correlation with subjective memory failures. Although the mean age of both samples is similar, the age range of PCC participants in Serrano del Pueblo *et al*.,^18^ is large, ranging from 23 to 72 years old, which could be an important factor to consider for interpreting the correlations and may be explaining the discrepancies with our study. A recent study including 223 subjects,^17^ assessed DTI changes using conventional DTI markers but also a fixel-based analysis to address more complex WM compositions. They found FA changes in COVID recovered patients in a voxel-wise approach showing increases and decreases in different regions, which may explain the lack of differences in whole-brain mean FA values between patients and controls. In fact, we also obtained the same negative result in our analysis. Even though results are not directly comparable due to the difference in our samples, since their participants were not PCC but individuals recovered from COVID-19 infection, their results highlight the importance of continuing research on microstructural WM changes in this condition and point towards the possible existence of different regional vulnerabilities or trajectories over time.

To elucidate structural brain changes in PCC patients with clinically meaningful cognitive impairment is of crucial interest considering it represents 51–58% of the active population who suffer from PCC^61^ and could be regarded as an at-risk population. Moreover, controversy on previous results based on cognitive impairment classification could open a debate about the clinical criteria used to identify cognitive impairment in PCC. When analysing structural brain changes in PCC patients divided according to the presence of cognitive impairment, we found significant cortical thickening in patients with clinical cognitive impairment in bilateral frontal brain areas, compared to non-cognitively altered patients. According to these results, higher cortical abnormalities are present in those PCC with cognitive impairment, compared to PCC patients without cognitive impairment. Additionally, differences in blood interleukin 6 and D-dimer levels were found between cognitively altered and non-cognitively altered patients. However, these differences disappeared after correcting for FDR. Future studies with larger PCC samples could be helpful to study the possible relationship between cognition and blood biomarkers, and its association with structural abnormalities.

Contrary to our CTh results, Serrano del Pueblo *et al*.,^18^ found no differences in CTh between cognitively altered and non-altered PCC participants. However, a different criterion was used to classify cognitively and non-cognitively altered PCC patients. While they divided patients into two groups according to the American Academy of Clinical Neuropsychology (overall cognitive level *z* ≤ −0.71 or overall cognitive level *z* > −0.71), we divided our patients according to the number of cognitive tests with *z* ≤ −1.5, derived from normative population data. We have used more restrictive criteria to consider PCC as cognitively altered, probably enhancing the cortical differences between both groups. Another study dividing PCC patients according to the presence of cognitive impairment, found higher CTh in both PCC groups, compared to HC.^19^ Even so, these cortical areas with increased thickness were anatomically more extended in PCC patients with cognitive impairment. In this study, PCC patients were classified as cognitively impaired if they presented a MoCA score <26, while in our study patients were classified according to the sum of altered cognitive tests from an extensive neuropsychological assessment. Their classification may be less restrictive than ours, perhaps including some patients in their non-altered group that would be considered cognitively altered individuals based on our criteria and highlighting the usefulness of comprehensive neuropsychological assessment to identify cognitive impairment in PCC as a possible indicator of more severe structural brain involvement.

Previous studies have suggested the effect of acute infection severity as a relevant factor in PCC sequelae,^15,62^ and hypoxia as a possible pathological underlying mechanism,^63,64^ among others. In this regard, we also investigated structural brain changes in PCC patients classified according to severity during COVID-19 acute infection. Regarding CTh, non-hospitalized PCC participants showed higher CTh in frontal regions and temporal regions, compared to HC and hospitalized PCC participants without ICU admission, respectively. Moreover, no differences were found in volumetric subcortical segmentations or GM VBM between groups.

The lack of results in hospitalized participants compared to HC, suggests that acute-infection severity may have a minimal effect on cortical structural brain abnormalities in this sample of PCC participants. Indeed, when analysis were performed including ICU participants in the hospitalized PCC group, the differences between hospitalized and non-hospitalized participants disappeared after adjusting for time interval between infection and the MRI acquisition dates. These results suggest that interval time since infection could be a better indicator of brain changes in these patients. Nevertheless, this hypothesis needs to be tested in longitudinal studies.

Concerning WM changes, higher volume of WMH was found in hospitalized PCC participants compared to non-hospitalized participants; however no significant differences were identified in FA values, as a marker of microstructural WM abnormalities, between PCC groups, or between hospitalized PCC and HC. Lower FA values were found only between non-hospitalized PCC and HC, indicating severity during the acute infection may not reflect microstructural WM abnormalities. Nevertheless, future analyses with larger sample sizes are necessary to confirm our results and to examine DTI changes in the ICU PCC participants. Due to the small size of this subgroup (*n* = 5), we were unable to perform between-group comparison analyses that included only the hospitalized ICU PCC group.

The main strength of the current work is the use of multimodal MRI approach to explore structural brain changes in an extensive post-COVID condition sample, subsequently characterized by the presence of clinical and cognitive impairment, in comparison to a group of HC. Moreover, in our study participants have performed a comprehensive neuropsychological battery, allowing us to study different cognitive domains, as well as to identify clinically meaningful cognitively impaired patients. However, this study had some limitations that should be considered. First, although our control participants had not tested positive for SARS-CoV-2 or shown compatible symptoms, we cannot rule out the possibility of having included individuals who were infected but remained asymptomatic. In this regard, we could not exclude participants depending on the presence of antibodies against the virus due to vaccination protocols. Moreover, local epidemiological data indicated that most participants were probably infected with the alpha, delta and omicron (BA. 1) variants. Therefore, we cannot address the effect of different variants in brain changes associated to COVID-19 sequalae. Another limitation regards the fact that sample size with diffusion-weighted images is slightly smaller than that with T1-weighted images, so results obtained from it should be taken with caution. Moreover, we did not have the neuropsychiatric questionnaires score from every PCC participant in the study, limiting the conclusion extracted from their association with brain abnormalities. Finally, as it is a cross-sectional study, the present work can only identify brain changes in patients with post-COVID compared to a control group, but not before COVID infection. Besides, the time interval between acute infection and the follow-up in our sample is highly heterogeneous, leading us to probably include PCC participants in different time points of the disease, and consequently with different cognitive and structural brain sequels, as we believe cognitive and brain changes in these patients could be dynamic across disease course. Hence, longitudinal designs in post-COVID condition will be useful to study trajectories and to clarify if brain and cognitive changes in PCC are dynamic. Further researches also need to be done with other imaging techniques to corroborate the underlying inflammatory process suggested from our results in PCC participants.

## Conclusion

In conclusion, our study identified structural brain alterations implying increased CTh and abnormal microstructural WM integrity, and worse cognitive performance across various cognitive domains in post-COVID condition patients almost 2 years after COVID infection. Changes in CTh are associated with clinically meaningful cognitive impairment while WM changes are related with subjective memory failures. These findings highlight the importance of monitoring the underlying pathological mechanisms of this condition and the significance of following this at-risk population in time.

## Supplementary Material

fcaf070_Supplementary_Data

## Data Availability

The data that support the findings of this study are available from the corresponding author upon reasonable request. The data are not publicly available due to information that could compromise the privacy of research participants.

## Appendix 1

Members of the NAUTILUS-project Collaborative Group: Vanesa Arauzo, Jose A. Bernia, Marta Balague-Marmaña, Berta Valles-Pauls, Jesús Caballero, Ester Gonzalez-Aguado, Carme Tayó-Juli, Eva Forcadell-Ferreres, Silvia Reverte-Vilarroya, Susanna Forné, Anna Bartes-Plans, Jordina Muñoz-Padros, Jose A. Muñoz-Moreno, Anna Prats-Paris, Inmaculada Rico, Nuria Sabé, Marta Almeria, Laura Casas, Maria José Ciudad, Anna Ferré, Tamar Garzon, Manuela Lozano, Marta Cullell, Sonia Vega, Sílvia Alsina, Maria J. Maldonado-Belmonte, Susana Vazquez-Rivera, Eva Baillès, Sandra Navarro, Ayoze González Hernández, Yaiza Molina, Victoria Olive, Silvia Cañizares.
