# Supplementary material for: Structural brain changes in post-COVID condition and its relationship with cognitive impairment
Source: Brain Commun. 2025 Feb 12;7(1):fcaf070. doi: 10.1093/braincomms/fcaf070 (PMC11851114; doi:10.1093/braincomms/fcaf070)
Supplement: fcaf070_Supplementary_Data [file fcaf070_supplementary_data.docx]

**SUPPLEMENTARY MATERIAL**

**Supplementary Table 1. Distribution of vaccinated data across participants.**

|  | | | |  | | | **HC (n=37)** | | **PCC (n=128)** | | | |
| --- | --- | --- | --- | --- | --- | --- | --- | --- | --- | --- | --- | --- |
| **Vaccinated, yes, n(%)** | | |  | | | 26 (70.3) | | 110 (85.9) | | | |  |
| Vaccinated before COVID-19 infection, n(%) | | | | - | | | 31 (24.2) | | | |  |  |
| Vaccinated after COVID-19 infection, n(%) | | | | - | | | 69 (53.9) | | | |  |  |
| NA | | | | - | | | 10 | | | |  |  |
| **Vaccinated, no, n(%)** | | | | | | 11 (29.7) | | 18 (14.1) | | | |  |
|  | | | |  | | |  | | **Non-hospitalized PCC (n=87)** | | **Hospitalized PCC (n=41)** | |
| **Vaccinated, yes, n(%)** | | | |  | | |  | | 78 (89.7) | 32 (78.0) | | |
| Vaccinated before COVID-19 infection, n(%) | | | |  | | | 29 (33.3) | 2 (4.9) | | |  |  |
| Vaccinated after COVID-19 infection, n(%) | | | |  | | | 43 (49.4) | 26 (63.4) | | |  |  |
| NA | | | |  | | | 6 | 4 | | |  |  |
| **Vaccinated, no, n(%)** | | | | | | |  | | 9 (10.3) | 9 (22.0) | | |
|  | | | |  | | |  | | **Non-altered PCC (n=57)** | | **Altered PCC (n=71)** | |
| **Vaccinated, yes, n(%)** | | | |  | | |  | | 49 (86.0) | 61 (85.9) | | |
| Vaccinated before COVID-19 infection, n(%) | | | |  | | | 15 (26.3) | 16 (22.5) | | |  |  |
| Vaccinated after COVID-19 infection, n(%) | | | |  | | | 30 (52.6) | 39 (54.9) | | |  |  |
| NA | | | |  | | | 4 | 6 | | |  |  |
| **Vaccinated, no, n(%)** | | | | | | |  | | 8 (14.0) | 10 (14.1) | | |

Abbreviations: HC, healthy controls; PCC, post-COVID condition; NA, non-answered date of vaccination.

**Supplementary Table 2. Previous comorbidities of PCC participants and healthy controls.**

|  | **n** | **HC** | **n** | **PCC** | **χ2 / p-value** |
| --- | --- | --- | --- | --- | --- |
| Hearth disease, n (%) | 26 | 0 (0.0) | 125 | 6 (7.5) | 1.30/.254 |
| Respiratory disease, n (%) | 26 | 0 (0.0) | 125 | 14 (17.5) | 3.21/.073 |
| Obesity, n (%) | 26 | 4 (1.04) | 125 | 29 (36.3) | 0.77/.380 |
| Dyslipidemia, n (%) | 26 | 6 (1.62) | 125 | 16 (20.0) | 1.83/.177 |
| Hypertension, n (%) | 26 | 2 (0.52) | 125 | 20 (25.0) | 1.19/.275 |
| Diabetes mellitus, n (%) | 26 | 3 (0.78) | 125 | 2 (2.5) | 6.64/.010* |

Abbreviations: HC, healthy controls; PCC, post-COVID condition. Group differences were analyzed with Pearson’s chi-squared test.

* p-value < 0.05.

**Supplementary Table 3. Distribution of blood biomarkers levels in PCC participants classified according to the presence of cognitive impairment.**

|  | **Altered PCC** | | **Non-altered PCC** | | **PCC** | |
| --- | --- | --- | --- | --- | --- | --- |
|  | **n** | **Mdn (min-max)** | **n** | **Mdn (min-max)** | **n** | **Mdn (min-max)** |
| CRP (mg/mL) | 31 | 20.18 (0.69-333.55) | 33 | 23.48 (3.71-189.05) | 64 | 23.39 (0.69-333.55) |
| IL-6 (pg/mL) | 31 | 2.36 (0.75-7.11) | 31 | 1.72 (0.77-5.08) | 62 | 1.88 (0.75-7.11) |
| NGF (pg/mL) | 30 | 3.08 (1.72-10.14) | 33 | 4.20 (2.31-10.11) | 63 | 3.59 (1.72-10.14) |
| Ferritin (ng/mL) | 31 | 41.80 (11.22-760.51) | 34 | 61.31 (10.53-456.02) | 65 | 44.50 (10.53-760.51) |
| Thrombomodulin  (ng/mL) | 32 | 2.99 (1.35-5.73) | 34 | 3.08 (1.78-7.06) | 66 | 2.99 (1.35-7.06) |
| Endothelin 1  (pg/mL) | 32 | 1.24 (0.58-1.91) | 34 | 1.11 (0.38-16.44) | 64 | 1.18 (0.38-16.44) |
| D-dimer (ng/mL) | 32 | 2956.99 (589.76-8435.11) | 34 | 4019.22 (595.79-25235.38) | 66 | 3357.90 (589.76-25235.38) |
| GFAP (pg/mL) | 32 | 51.75 (12.24-199.85) | 34 | 58.84 (29.22-174.73) | 66 | 55.56 (12.24-199.85) |
| MDA (pg/mL) | 32 | 50.79 (24.69-147.98) | 34 | 46.28 (29.80-284.69) | 66 | 47.28 (24.69-284.69) |

Abbreviations: CRP, C Reactive Protein; GFAP, Glial fibrillary acidic protein; IL-6, Interleukin 6; max, màximum; MDA, Malondialdehyde; mdn, median; min, mínimum; NGF, Nerve Growth Factor; PCC, Post-COVID condition,

**Supplementary Table 4. Significant clusters of cortical thickness analysis between PCC and HC participants.**

| **Cluster Wise (mm2)** | | **MNI305 space** | | | | | **Clusterwise p-value** | **Cluster anatomical annotation** |
| --- | --- | --- | --- | --- | --- | --- | --- | --- |
|  | | **X** | | **Y** | | **Z** |  |  |
| PCC > HC | | | | | | | | |
| RH clusters | |  |  | |  | |  |  |
| 1 | 2572.68 | 8.6 | 24.3 | | 48.0 | | 0.00080 | Superior frontal gyrus |
| 2 | 2234.87 | 37.7 | 32.0 | | 12.1 | | 0.00360 | Rostral middle frontal gyrus |

Abbreviation: HC, healthy controls; PCC, post-COVID condition; RH; right hemisphere.

**Supplementary Table 5. Volumetric measures of subcortical structures differences between PCC and control participants.**

|  | **HC (n=37)** | **PCC (n=128)** |  | |
| --- | --- | --- | --- | --- |
| **Volume (cm^3^)** | **Mdn (min-max)** | **Mdn (min-max)** | **Test stat/ p-value** | |
| Left lateral ventricle | 7.74 (3.35 – 28.60) | 7.94 (2.01 – 29.69) | 0.009/>.99 |  |
| Left inferior lateral ventricle | 0.29 (0.06 – 0.60) | 0.26 (0.04 – 1.18) | 0.95/.997 |  |
| Left cerebellum WM | 14.18 (9.56 – 22.67) | 13.67 (10.29 - 20.14) | 0.47/>.99 |  |
| Left cerebellum cortex | 54.45 (42.88 – 79.27) | 52.59 (39.49 – 70.90) | 1.17/.990 |  |
| Left thalamus | 7.54 (5.63 – 9.96) | 7.27 (5.70 – 9.99) | 0.26/>.99 |  |
| Left caudate | 3.48 (2.51 – 4.64) | 3.29 (2.08 – 4.21) | 2.11/.878 |  |
| Left putamen | 4.66 (2.68 – 5.78) | 4.51 (3.40 - 5.98) | 0.85/>.99 |  |
| Left pallidum | 1.99 (1.46 – 2.71) | 1.95 (1.48 – 2.79) | 0.16/>.99 |  |
| 4th ventricle | 1.68 (0.95 – 2.97) | 1.69 (0.88 – 3.14) | 0.27/>.99 |  |
| Brain stem | 21.33 (15.5 – 32.66) | 20.94 (15.77 – 28.78) | 0.62/>.99 |  |
| Left hippocampus | 4.03 (2.95 – 5.06) | 3.89 (3.18 – 4.90) | 1.32/.985 |  |
| Left amygdala | 1.50 (1.17 – 1.92) | 1.49 (1.11 – 2.21) | 0.012/>.99 |  |
| CSF | 1.02 (0.58 – 1.73) | 0.91 (0.55 – 1.62) | 0.93/.997 |  |
| Left accumbens area | 0.41 (0.22 – 0.55) | 0.38 (0.19 – 0.61) | 1.81/.927 |  |
| Left ventral diencephalon | 4.12 (3.14 – 5.41) | 3.85 (3.04 – 5.27) | 4.75/.356 |  |
| Left vessel | 0.04 (0.007- 0.08) | 0.03 (0.003 – 0.17) | 1.11/.992 |  |
| Left choroid plexus | 0.51 (0.16 – 0.80) | 0.47 (0.18 – 1.09) | 0.02/>.99 |  |
| Right lateral ventricle | 7.12 (2.80 – 20.79) | 7.46 (2.08 – 26.62) | 0.27/>.99 |  |
| Right inferior lateral ventricle | 0.27 (0.14 – 0.75) | 0.28 (0.07 – 0.96) | 0.02/>.99 |  |
| Right cerebellum WM | 14.06 (10.44 – 22.05) | 13.17 (9.50 – 19.76) | 2.94/.730 |  |
| Right cerebellum cortex | 55.00 (44.78 - 82.58) | 54.02 (40.14 – 70.61) | 0.94/.997 |  |
| Right thalamus | 7.34 (5.66 – 9.46) | 7.15 (5.53 – 9.44) | 0.18/>.99 |  |
| Right caudate | 3.56 (2.46 - 4.66) | 3.42 (2.48 – 4.98) | 0.92/.997 |  |
| Right putamen | 4.74 (3.79 – 5.78) | 4.65 (3.54 – 6.07) | 0.61/>.99 |  |
| Right pallidum | 1.94 (1.43 – 2.78) | 1.90 (1.38 – 2.56) | 0.02/>.99 |  |
| Right hippocampus | 4.09 (3.23 – 5.15) | 4.07 (3.14 – 5.10) | 0.39/>.99 |  |
| Right amygdala | 1.69 (1.33 – 2.18) | 1.60 (1.16 – 2.17) | 1.10/.993 |  |
| Right accumbens area | 0.55 (0.38 – 0.75) | 0.51 (0.33 – 0.72) | 4.13/.444 |  |
| Right ventral diencephalon | 4.03 (3.11 – 5.24) | 3.82 (3.05 – 5.28) | 3.93/.475 |  |
| Right vessel | 19.40 (2.60 - 82.40) | 18.10 (0.00 - 189.60) | 0.06/>.99 |  |
| Right choroid plexus | 0.53 (0.14 – 0.91) | 0.48 (0.21 – 1.45) | 1.05/.994 |  |
| Optic chiasm | 0.20 (0.14 – 0.25) | 0.19 (0.03 – 0.28) | 0.21/>.99 |  |
| CC posterior | 1.01 (0.72 – 1.33) | 0.98 (0.55 – 1.45) | 0.70/>.99 |  |
| CC mid posterior | 0.57 (0.35 – 0.85) | 0.55 (0.27 – 0.97) | 0.76/>.99 |  |
| CC central | 0.56 (0.38 – 0.92) | 0.54 (0.32 – 0.98) | 2.31/.834 |  |
| CC mid anterior | 0.53 (0.36 – 0.85) | 0.51 (0.30 – 0.99) | 1.20/.988 |  |
| CC anterior | 0.90 (0.62 – 1.30) | 0.87 (0.54 – 1.30) | 0.26/>.99 |  |
| Cortex volume | 456.41 (338.35 – 581.21) | 449.38 (339.03 – 552.80) | 0.006/>.99 |  |
| Cerebral WM volume | 453.81 (318.62 – 616.29) | 437.93 (297.69 – 588.95) | 3.02/.715 |  |
| Subcortical gray volume | 57.17 (44.84 – 70.17) | 54.92 (45.01 – 67.52) | 1.20/.988 |  |
| Total gray volume | 625.95 (476.69 – 778.59) | 615.17 (475.95 – 735.60) | 0.003/>.99 |  |

Abbreviations: CC, corpus callosum; CSF, cerebrospinal fluid; HC, healthy controls; max, maximum; mdn, median; min, minimum; PCC, post-COVID condition; WM, white matter. Group differences were tested using a GLM.

**Supplementary Table 6. Demographic characteristics of PCC participants and healthy controls in the subsample included in the DTI analyses.**

|  | **HC (n = 37)** | **PCC (n = 98)** | **Test stat/ p-value** |
| --- | --- | --- | --- |
| Age in years, mdn (min-max) | 54.00 (41-61) | 51.50 (31-62) | -0.82/.476 |
| Education in years, mdn (min-max) | 16.00 (9-21) | 15.00 (9-20) | -0.22/.884 |
| Sex, female, n (%) | 25 (67.6) | 86 (87.70) | 7.49/.030** |
| IQ, mdn (min-max) | 107.00 (98-114) | 106.00 (85-116) | -1.06/.291 |
| Hospitalized, n (%) | - | 15 (15.31) | - |
| ICU, n (%) † | - | 5 (5.10) | - |
| Vaccine, yes, n (%) | 26 (70.27) | 88 (89.80) | 2.87/.223 |
| Interval COVID-MRI in months, mdn (min-max) | - | 20.00 (3-44) | - |
| Interval NP-MRI in months, mdn (min-max) | 0.00 (0-3) | 0.00 (0-5) | -0.47/.641 |

Abbreviations: HC, healthy controls; ICU, intensive care unit; IQ, intelligence quotient; max, maximum; mdn, median; min, minimum; MRI, magnetic resonance imaging; NP, neuropsychological assessment. Group differences were tested using independent Mann-Whitney U. Differences in categorical variables were analyzed with Pearson’s chi-squared test.

** p-value < 0.05 FDR corrected.

 † ICU admitted participants are included in the total number of hospitalizations.

**Supplementary Table 7. Differences in neuropsychological assessment of PCC participants and healthy controls in the subsample included in the DTI analyses**.

|  | **HC** | | **PCC** | |  |
| --- | --- | --- | --- | --- | --- |
|  | **n** | **Mdn (min-max)** | **n** | **Mdn (min-max)** | **F/ p** |
| **MoCA** | 26 | 28 (25-30) | 90 | 27 (18-30) | 2.28/.036** |
| **Matrix** | 26 | 20 (5-26) | 98 | 19 (6-50) | 0.09/.648 |
| **RAVLT Total** | 37 | 50 (35-65) | 98 | 47 (25-65) | 4.22/.013** |
| **RAVLT delayed recall** | 37 | 10 (4-15) | 98 | 9 (1-15) | 4.95/.010** |
| **RAVLT recognition** | 37 | 14 (10-15) | 97 | 13 (3-15) | 3.49/.010** |
| **ROCF immediate memory accuracy** | 26 | 20.5 (8.5-30) | 60 | 20 (1-85) | 0.09/.671 |
| **ROCF delayed memory accuracy** | 26 | 21 (8-29) | 60 | 20 (1-30) | 0.06/.708 |
| **Digit span forward** | 26 | 6 (4-8) | 98 | 6 (3-9) | 4.05/.006** |
| **Digit span backwards** | 26 | 5 (4-8) | 98 | 4 (2-8) | 5.55/.006** |
| **DSC coding** | 26 | 77 (37-100) | 97 | 66 (32-100) | 4.76/.008** |
| **TMTA** | 37 | 27 (15-76) | 98 | 35 (16-135) | 2.04/.044** |
| **TMTB** | 37 | 66 (31-170) | 98 | 71 (10-276) | 0.75/.215 |
| **Stroop W** | 35 | 106 (62-131) | 98 | 95 (20-144) | 4.94/.005** |
| **Stroop C** | 35 | 70 (46-92) | 98 | 61 (21-94) | 6.57/.003** |
| **Stroop WC** | 35 | 42 (12-65) | 98 | 37.5 (13-96) | 1.20/.111 |
| **Phonemic fluency (PMR)** | 26 | 48.5 (23-77) | 97 | 39 (14-72) | 9.68/.001** |
| **Semantic fluency (Animals)** | 37 | 24 (11-35) | 98 | 19.5 (8-38) | 3.80/.015** |
| **BNT** | 37 | 55 (14-60) | 61 | 54 (47-59) | 1.17/.145 |
| **RMET** | 26 | 24 (15-28) | 61 | 23 (17-31) | 1.49/.145 |
| **UPSIT** | 32 | (27-38) | 43 | 30 (11-37) | 5.05/.001** |

Abbreviations: BNT, Boston Naming Test; DSC, Digit Symbol Coding; HC, healthy controls; max, maximum; mdn, median; min, minimum; MoCA, Montreal Cognitive Assessment; PCC, post-COVID condition; PMR: phonemic fluency; RAVLT, Rey’s Auditory Verbal Learning Test; RAVLT delayed recall, total recall after 20 min; RAVLT total, sum of correct responses from trial I to trial V; RMET, Reading the Mind in the Eyes Test; ROFC, Rey–Osterrieth Complex Figure Test; Stroop W, Stroop Words; Stroop C, Stroop Colors; Stroop WC, Stroop Words-Colors; TMTA, Trail Making Test part A; TMTB, Trail Making Test part B. UPSIT, University of Pennsylvania Smell Identification Test. Group differences were tested using GLM.

** p-value < 0.05 FDR corrected.

**Supplementary Table 8. Clinical characteristics between PCC and healthy controls.**

|  | **HC** | | **PCC** | |  |
| --- | --- | --- | --- | --- | --- |
|  | **n** | **Mdn (min-max)** | **n** | **Mdn (min-max)** | **Test stats/ p -value** |
| CFQ, mdn (min-max) | 24 | 1.5 (0-10) | 86 | 10 (0-11) | 50.87/.001** |
| GAD-7, mdn (min-max) | 24 | 3 (0-13) | 86 | 6 (0-21) | 5.58/.001** |
| PHQ-9, mdn (min-max) | 24 | 3 (0-9) | 87 | 11 (0-25) | 17.02/.001** |
| MFE, mdn (min-max) | 24 | 7 (1-22) | 87 | 27 (0-51) | 23.1/.001** |

Abbreviations: CFQ, Chalder Fatigue Scale; GAD-7, Generalized Anxiety Disorder 7-item scale; HC, healthy controls; max, maximum; mdn, median; MFE, Memory Failures of Everyday; min, minimum; PCC, Post-COVID condition; PHQ-9, Patient Health Questionnaire-9. Group differences were tested using GLM.

**p-value < 0.05, FDR corrected

**Supplementary Table 9. Fractional anisotropy differences between PCC and healthy controls.**

| **FA PCC < HC** | | | | | | |
| --- | --- | --- | --- | --- | --- | --- |
| Clusters | Voxels | Max | Max X (mm) | Max Y (mm) | Max Z (mm) | Region |
| 10 | 3192 | 0.960 | 39 | -52 | 15 | Superior longitudinal fasciculus R |
| 9 | 2705 | 0.962 | -9 | -33 | 21 | Splenium of corpus callosum |
| 8 | 293 | 0.952 | 18 | 22 | 24 | Genu of corpus callosum |
| 7 | 154 | 0.952 | 37 | -55 | 33 | 5% of superior longitudinal fasciculus R |
| 6 | 112 | 0.951 | 43 | 9 | -24 | 20% uncinate fasciculus R |
| 5 | 91 | 0.951 | 15 | 36 | -12 | 11% uncinate fasciculus R |
| 4 | 72 | 0.951 | 20 | 44 | -1 | 29% forceps major |
| 3 | 71 | 0.952 | 58 | -19 | -13 | - |
| Abbreviation: FA, fractional anisotropy; HC, healthy controls; PCC, post-COVID condition; R, right. Just clusters with a number of voxels bigger than 50 are reported. Location of the significant results were obtained with JHU atlases.      **Supplementary Table 10. Whole-brain mean diffusion parameters differences between PCC and healthy controls.**   \|  \| **HC (n=37)** \| **PCC (n= 98)** \| **Test stat/ p-value** \| \| --- \| --- \| --- \| --- \| \| Mean FA, mdn (min-max) \| 0.462 (0.41-0.49) \| 0.459 (0.42-0.51) \| 2.76/.016* \| \| Mean MD, mdn (min-max) \| 6.3·10-4 (6·10-4 - 7·10-4) \| 6.2·10-4 (6·10-4 - 7·10-4) \| 0.41/.366 \| \| Mean RD, mdn (min-max) \| 4.5·10-4 (4·10-4 - 5·10-4) \| 4.6·10-4 (4·10-4 - 5·10-4) \| 1.45/.111 \| \| Mean AD, mdn (min-max) \| 9.7·10-4 (9·10-4 - 10·10-4) \| 9.8·10-4 (9·10-4 - 10·10-4) \| 0.10/.636 \|   Abbreviation: AD, axial diffusivity; FA, fractional anisotropy; HC, healthy controls; max, maximum; MD, mean diffusivity; mdn, median; min, minimum; PCC, post-COVID condition; RD, radial diffusivity. Group differences were tested using GLM.  * p-value < 0.05 | | | | | | |

**Supplementary Table 11. Demographic characteristics of PCC participants classified according to the presence of cognitive impairment.**

|  | **Altered PCC (n= 71)** | **Non-altered PCC (n=57)** | **Test stat/ p-value** |
| --- | --- | --- | --- |
| Age in years, mdn (min-max) | 50.00 (31-62) | 54.00 (30-65) | -3.44/.001* |
| Education years, mdn (min-max) | 14.00 (8-20) | 17.00 (9-24) | -2.25/.025* |
| Sex, female, n(%) | 54 (76.05) | 44 (77.19) | 0.23/.880 |
| IQ, mdn (min-max) | 104.00 (85-116) | 108.00 (89-116) | -3.46/.001* |
| Hospitalized, n(%) | 25 (35.21) | 16 (28.07) | 0.74/.389 |
| ICU, n(%) † | 15 (21.13) | 9 (15.79) | 0.80/.672 |
| Interval COVID-MRI in months, mdn (min-max) | 18.00 (3-44) | 16.00 (3-44) | -0.77/.441 |
| Interval NP-MRI in months, mdn (min-max) | 0.00 (0-5) | 0.00 (0-4) | -0.39/.699 |

Abbreviations: HC, healthy controls; ICU, intensive care unit; IQ, intelligence quotient; max, maximum; mdn, median; min, minimum; MRI, magnetic resonance imaging; NP, neuropsychological assessment; PCC, post-COVID condition. Statistically significant differences (p<0.05) indicated in bold. Group differences were tested using Kruskal Wallis. Differences in categorical variables were analyzed with Pearson’s chi-squared test.

* p-value < 0.05.

 † ICU admitted participants are included in the total number of hospitalizations.

**Supplementary Table 12. Clinical characteristics of PCC participants classified based on the presence of cognitive impairment.**

|  | **Altered PCC** | | **Non-altered PCC** | |  |
| --- | --- | --- | --- | --- | --- |
|  | **n** | **Mdn (min-max)** | **n** | **Mdn (min-max)** | **Test stat/ p-value** |
| CFQ | 64 | 10.00 (0-11) | 50 | 10.00 (0-11) | 2.05/.278 |
| GAD-7 | 64 | 5.50 (0-21) | 50 | 5.00 (0-19) | 0.44/.732 |
| PHQ-9 | 65 | 11.00 (0-22) | 50 | 8.50 (0-25) | 2.80/.101 |
| MFE | 64 | 27.00 (0-51) | 51 | 20.00 (0-48) | 4.16/.188 |

Abbreviations: CFQ, Chalder Fatigue Scale; GAD-7, Generalized Anxiety Disorder 7-item scale; max, maximum; mdn, median; MFE, Memory Failures of Everyday; min, minimum; PCC, post-COVID condition; PHQ-9, Patient Health Questionnaire-9. Group differences were tested using GLM.

**Supplementary Table 13. Blood biomarkers differences between PCC participants classified according to the presence of cognitive impairment.**

|  |  | **Altered PCC** |  | **Non-altered PCC** |  |
| --- | --- | --- | --- | --- | --- |
|  | **n** | **Mdn (min-max)** | **n** | **Mdn (min-max)** | **Test stat/ p** |
| CRP (mg/mL) | 31 | 20.18 (0.69-333.55) | 33 | 23.48 (3.71-189.05) | 0.00/.983 |
| IL-6 (pg/mL) | 31 | 2.36 (0.75-7.11) | 31 | 1.72 (0.77-5.08) | 4.93/.032* |
| NGF (pg/mL) | 30 | 3.08 (1.72-10.14) | 33 | 4.20 (2.31-10.11) | 2.09/.158 |
| Ferritin (ng/mL) | 31 | 41.80 (11.22-760.51) | 34 | 61.31 (10.53-456.02) | 3.64/.060 |
| Thrombomodulin  (ng/mL) | 32 | 2.99 (1.35-5.73) | 34 | 3.08 (1.78-7.06) | 3.29/.422 |
| Endothelin 1  (pg/mL) | 32 | 1.24 (0.58-1.91) | 34 | 1.11 (0.38-16.44) | 0.38/.981 |
| D-dimer (ng/mL) | 32 | 2956.99 (589.76-8435.11) | 34 | 4019.22 (595.79-25235.38) | 3.95/.047* |
| GFAP (pg/mL) | 32 | 51.75 (12.24-199.85) | 34 | 58.84 (29.22-174.73) | -0.49/.998 |
| MDA (pg/mL) | 32 | 50.79 (24.69-147.98) | 34 | 46.28 (29.80-284.69) | 1.96/.669 |

Abbreviations: CRP, C Reactive Protein; GFAP, Glial fibrillary acidic protein; IL-6, Interleukin 6; max, maximum; MDA, Malondialdehyde; mdn, median; min, minimum; NGF, Nerve Growth Factor; PCC, Post-COVID condition, Group differences were tested using GLM.

*p-value < 0.05

** p-value < 0.05 FDR corrected

**Supplementary Table 14. Significant clusters of cortical thickness analysis between altered PCC and non-altered PCC participants.**

| **Cluster Wise (mm2)** | | **MNI305 space** | | | | | **Clusterwise p-value** | **Cluster anatomical annotation** |
| --- | --- | --- | --- | --- | --- | --- | --- | --- |
|  | | X | | Y | | Z |  |  |
| **Altered PCC > Non-altered PCC** | | | | | | | | |
| LH clusters | |  |  | |  | |  |  |
| 1 | 1537.64 | -24.4 | 47.5 | | 13.1 | | 0.04371 | Rostral middle frontal gyrus |
| RH clusters | | | | | | | | |
| 1 | 2215.39 | 9.0 | 58.1 | | -3.1 | | 0.00360 | Medial orbito frontal |

Abbreviation: PCC, post-COVID condition; LH, left hemisphere; RH, right hemisphere.

**Supplementary Table 15.** **Demographic characteristics of PCC participants classified according to the severity during the acute infection and healthy controls.**

|  | **HC (n=37)** | **Non-hospitalized PCC (n=87)** | **Hospitalized PCC (n=17)** | **Hospitalized-ICU PCC (n=24)** | **Test stat/ p-value** |
| --- | --- | --- | --- | --- | --- |
| Age in years, mdn (min-max) | 54.00 (41-61) | 52.00 (30-65) | 52.00 (42-62) | 53.00 (42-59) | 2.19/.534 |
| Education years, mdn (min-max) | 16.00 (9-21) | 15.00 (9-24) | 12.00 (8-20) | 12.00 (9-19) | 10.50/.015^cf^ |
| Gender, female, n (%) | 25 (67.6) | 75 (86.2) | 12 (70.59) | 11 (45.83) | 17.75/.000^af^ |
| IQ, mdn (min-max) | 107.00 (98-114) | 107.00 (85-116) | 104.00 (85-116) | 103.00 (85-116) | 5.97/.113 |
| Interval COVID-MRI in months, mdn (min-max) | - | 20.00 (3-44) | 17.00 (4-39) | 8.00 (3-42) | 19.53/.001^ef^ |
| Interval NP-MRI in months, mdn (min-max) | 0.00 (0-3) | 0.00 (0-5) | 1.00 (0-4) | 0.00 (0-2) | 17.87/.001^bde^ |

Abbreviations: HC, healthy controls; ICU, intensive care unit; IQ, intelligence quotient; max, maximum; mdn, median; min, minimum; MRI, magnetic resonance imaging; NP, neuropsychological assessment; PCC, post-COVID condition. Group differences were tested using Kruskal-Wallis. Differences in categorical variables were analyzed with Pearson’s chi-squared test.

^a^ p-value between HC and non-hospitalized PCC <0.05.

^b^ p-value between HC and hospitalized PCC <0.05.

^c^ p-value between HC and ICU PCC <0.05.

^d^ p-value between hospitalized and non-hospitalized PCC <0.05.

^e^ p-value between hospitalized and ICU PCC <0.05.

^f^ p-value between ICU and non-hospitalized PCC <0.05.

**Supplementary Table 16. Demographic characteristics of PCC participants classified according to the severity during the acute infection, including hospitalized-ICU participants in hospitalized group, and healthy controls.**

|  | **HC (n=37)** | **Non-hospitalized PCC (n=87)** | **Hospitalized PCC (n=41)** | **Test stat/ p-value** |
| --- | --- | --- | --- | --- |
| Age in years, mdn (min-max) | 54.00 (41-61) | 52.00 (30-65) | 52.00 (42-62) | 1.29/.256 |
| Education years, mdn (min-max) | 16.00 (9-21) | 15.00 (9-24) | 12.00 (8-20) | 10.36/.001^bc^ |
| Gender, female, n (%) | 25 (67.6) | 75 (86.2) | 23 (56.1) | 14.54/.001^ac^ |
| IQ, mdn (min-max) | 107.00 (98-114) | 106.00 (87-116) | 104.00 (85-116) | 0.67/.413 |
| ICU, n (%) | - | - | 24 (58.54) | - |
| Interval COVID-MRI in months, mdn (min-max) | - | 20.00 (3-44) | 13.00 (3-42) | 14.95/.000^c^ |
| Interval MRI-NPS in months, mdn (min-max) | 0.00 (0-3) | 0.00 (0-5) | 0.00 (0-5) | 2.35/.309 |

Abbreviations: HC, healthy controls; ICU, intensive care unit; IQ, intelligence quotient; max, maximum; mdn, median; min, minimum; MRI, magnetic resonance; NP, neuropsychological assessment; PCC, post-COVID condition. Group differences were tested using Kruskal-Wallis. Differences in categorical variables were analyzed with Pearson’s chi-squared test.

^a^ p-value between HC and non-hospitalized PCC <0.05.

^b^ p-value between HC and hospitalized PCC <0.05.

^c^ p-value between hospitalized and non-hospitalized PCC <0.05.

**Supplementary Table 17. Demographic characteristics of PCC participants classified according to the severity during the acute infection and healthy controls, in the subsample included in the DTI analyses.**

|  | **HC (n=37)** | **Non-hospitalized PCC (n=83)** | **Hospitalized PCC (n=15)** | **Test stat/ p-value** |
| --- | --- | --- | --- | --- |
| Age in years, mdn (min-max) | 54.00 (41-61) | 52.00 (30-65) | 52.00 (43-62) | 1.13/.569 |
| Education years, mdn (min-max) | 16.00 (9-21) | 15.00 (9-24) | 13.00 (8-17) | 3.98/.137 |
| Gender, female, n (%) | 25 (67.6) | 74 (89.2) | 12 (80.0) | 8.22/.016 ^a^ |
| IQ, mdn (min-max) | 107.00 (98-114) | 107.00 (85-116) | 104.00 (85-114) | 5.34/.069 |
| ICU, n (%) | - | - | 5 (29.41) |  |
| Interval COVID-MRI in months, mdn (min-max) | - | 20.00 (3-44) | 18.50 (4-42) | -0.82/.411 |
| Interval NP-MRI in months, mdn (min-max) | 0.00 (0-3) | 0.00 (0-5) | 1 (0-4) | 7.86/.020 ^a^ |

Abbreviations: HC, healthy controls; ICU, intensive care unit; IQ, intelligence quotient; max, maximum; mdn, median; min, minimum; MRI, magnetic resonance imaging; NP, neuropsychological assessment; PCC, post-COVID condition. Group differences were tested using Kruskal-Wallis. Differences in categorical variables were analyzed with Pearson’s chi-squared test.

^a^ p-value between HC and mild PCC <0.05.

^b^ p-value between HC and severe PCC <0.05.

^c^ p-value between mild and severe PCC <0.05.


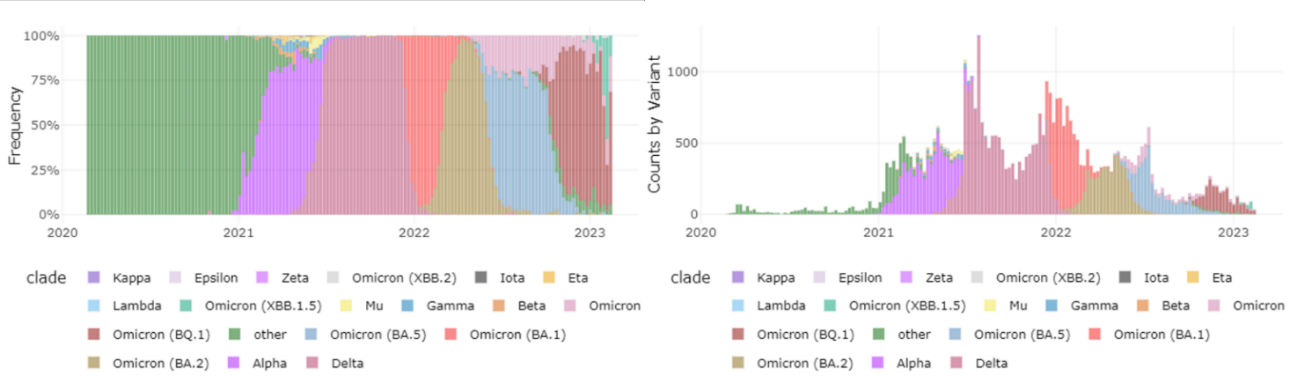


**Supplementary Figure 1. Average weekly Sars-CoV-2 variants in Catalonia from February 2020 to February 2023.** Frequency and counts by Sars-CoV-2 variant are reported. Variants are classified according to World Health Organization. Data and figures obtained from CovidTag http://covidtag.paseq.org (Enabled by Data from GISAID <https://www.gisaid.org/>).


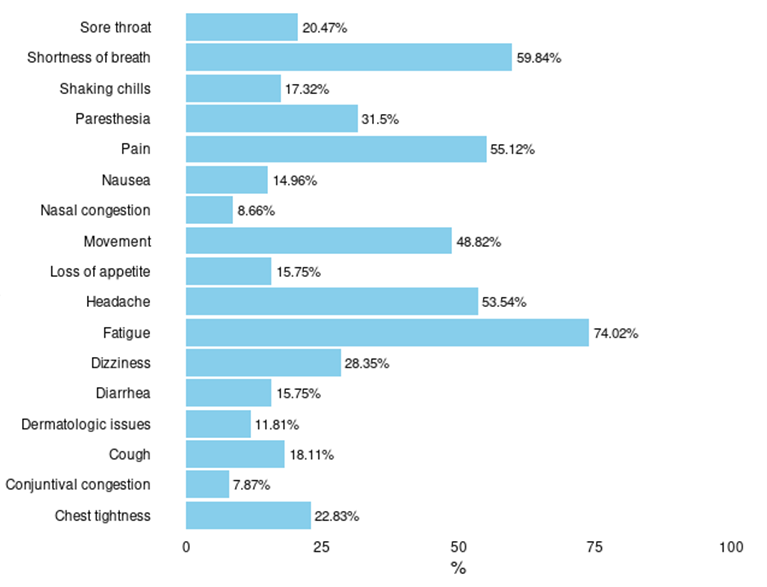


**Supplementary Figure 2. Frequency of sequelae symptoms in post-COVID patients at time of evaluation.** Frequencies are presented as percentage in post-COVID group.


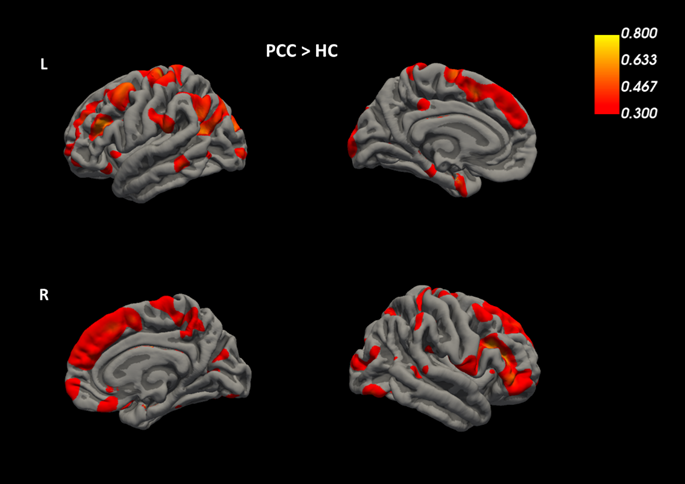


**Supplementary Figure 3. Effect sizes of group comparisons between PCC and HC groups.** Cohen’s d effect sizes (d > 0.3) are presented as absolute values and indicate higher magnitude of the difference between groups. Yellow color indicates larger differences between groups. Abbreviations: HC, healthy controls; L, left; PCC, post-COVID condition; R, right.


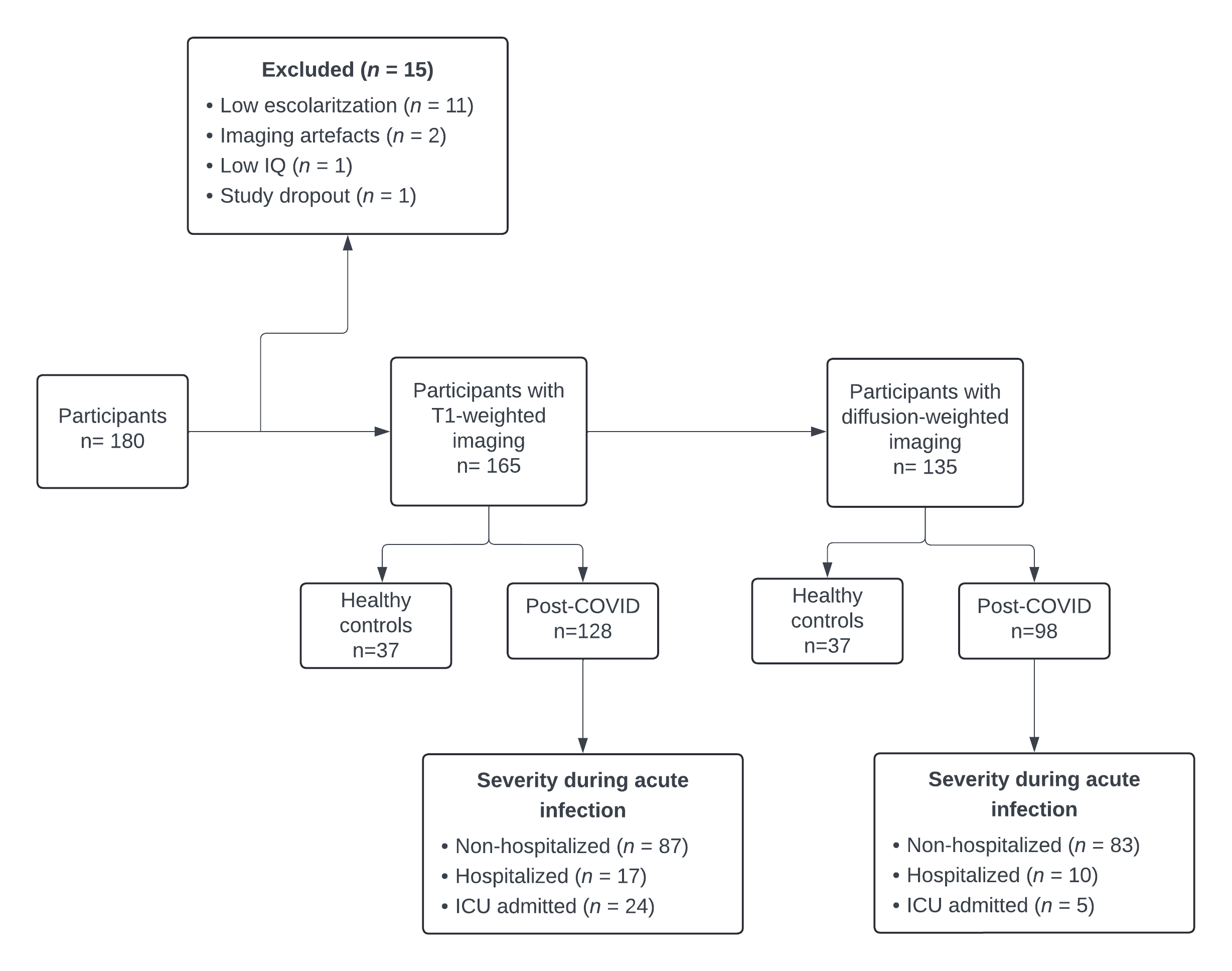


**Supplementary Figure 4. Flowchart with detailed steps until the ﬁnal sample composition regarding MRI acquisition.** Abbreviations: IQ, intelligent quotient; ICU, Intensive Care Unit. Created with https://lucid.app/.


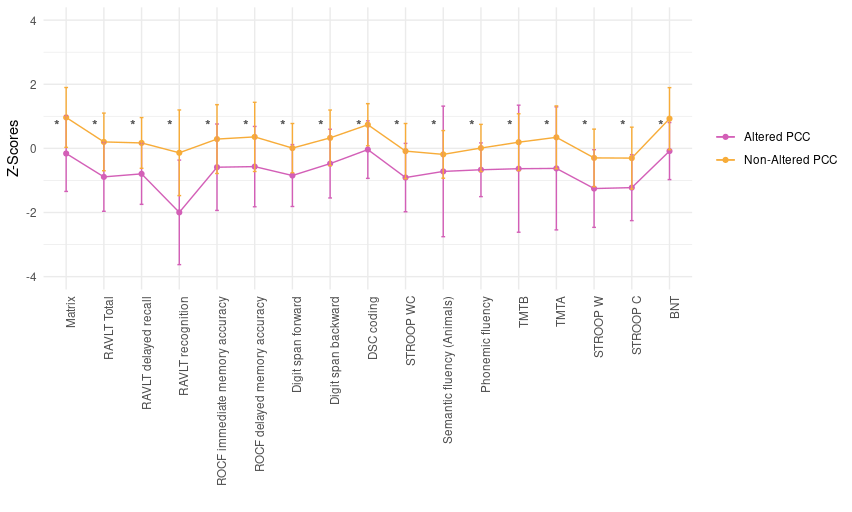


**Supplementary Figure 5. Cognitive profiles of post-COVID condition participants**. Cognitively altered PCC are presented in pink and non-altered PCC in orange. Data are presented as means of z-scores. Lower z-scores indicate poorer performance. Abbreviations: BNT, Boston Naming Test; DSC, Digit Symbol Coding; PCC, post-COVID condition; RAVLT, Rey’s Auditory Verbal Learning Test; RAVLT delayed recall, total recall after 20 min; RAVLT total, sum of correct responses from trial I to trial V; ROFC, Rey–Osterrieth Complex Figure Test; Stroop W, Stroop Words; Stroop C, Stroop Colors; Stroop WC, Stroop Words-Colors; TMTA, Trail Making Test part A; TMTB, Trail Making Test part B.

* p-value < 0.05 FDR corrected.


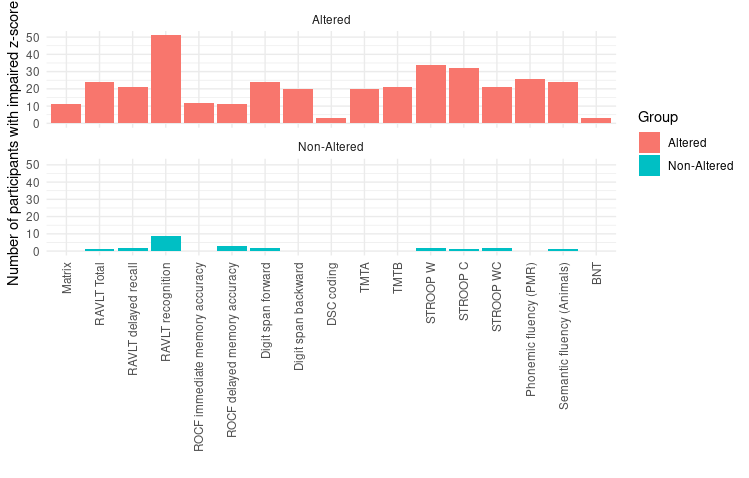


**Supplementary Figure 6. Frequency of PCC participants with z-score ≤ -1.5 for each cognitive test.** The bar graphic shows the number of individuals with a z-score ≤ -1.5 for each cognitive test, in altered and non-altered PCC groups. Abbreviations: BNT, Boston Naming Test; DSC, Digit Symbol Coding; RAVLT, Rey’s Auditory Verbal Learning Test; RAVLT delayed recall, total recall after 20 min; RAVLT total, sum of correct responses from trial I to trial V; ROFC, Rey–Osterrieth Complex Figure Test; Stroop W, Stroop Words; Stroop C, Stroop Colors; Stroop WC, Stroop Words-Colors; TMTA, Trail Making Test part A; TMTB, Trail Making Test part B.

**Supplementary Information 1.** **Members of the NAUTILUS-project collaborative group – List of Authors and Affiliations**

Vanesa Arauzo and Jose A. Bernia, Consorci Sanitari de Terrassa (CST), Terrassa, Spain. Marta Balague-Marmaña and Berta Valles-Pauls, Hospital Sant Joan Despí Moisès Broggi, Consorci Sanitari Integral. Jesús Caballero, Hospital Universitari Arnau de Vilanova, Lleida, Spain. Ester Gonzalez-Aguado and Carme Tayó-Juli, Consorci Sanitari Alt Penedès-Garraf, Vilafranca de Penedés, Barcelona, Spain. Eva Forcadell-Ferreres and Silvia Reverte-Vilarroya, Hospital Verge de la Cinta, Tortosa, Tarragona, Spain. Susanna Forné, Fundació Sant Hospital de la Seu d’Urgell, La Seu d’Urgell, Lleida, Spain. Anna Bartes-Plans and Jordina Muñoz-Padros, Consorci Hospitalari de Vic, Vic, Barcelona, Spain. Jose A. Muñoz-Moreno and Anna Prats-Paris, Servei de Malalties Infeccioses, Fundació Lluita contra les Infeccions – Hospital Universitari Germans Trias i Pujol, Badalona, Barcelona, Spain. Inmaculada Rico and Nuria Sabé, Hospital Universitari de Bellvitge, L’Hospitalet de Llobregat, Barcelona, Spain. Marta Almeria and Laura Casas, Hospital Universitari Mútua Terrassa, Terrassa, Barcelona, Spain. Maria José Ciudad and Anna Ferré, Badalona Serveis Assistens, Badalona, Barcelona, Spain. Tamar Garzon and Manuela Lozano, Institut d’Assistència Sanitària, Girona, Spain. Marta Cullell and Sonia Vega, Fundació Salut Empordà, Figueres, Girona, Spain. Sílvia Alsina, Fundació Hospital de Puigcerdà, Puigcerdà, Girona, Spain. Maria J. Maldonado-Belmonte and Susana Vazquez-Rivera, Hospital Universitario Central de la Cruz Roja San José y Santa Adela, Madrid, Spain. Eva Baillès and Sandra Navarro, Servei Andorrà d’Atenció Sanitària (SAAS), Andorra. Ayoze González Hernández, Facultad de Ciencias de la Salud, Universidad Fernando Pessoa Canarias. Yaiza Molina, Clínica Universitaria de Psicología, Facultad de Ciencias de la Salud, Universidad Fernando Pessoa Canarias. Victoria Olive, Occupational Health Care Service, Hospital Clínic Barcelona. Silvia Cañizares, Section of Clinical Psychology of Health, Clinical Institute of Neurosciences, Hospital Clinic of Barcelona. Department of Clinical Psychology and Psychobiology, Universitat de Barcelona.
